# Supplementary material for: Phenotypic heterogeneity follows a growth-viability tradeoff in response to amino acid identity
Source: Nat Commun. 2024 Aug 2;15:6515. doi: 10.1038/s41467-024-50602-8 (PMC11297284; doi:10.1038/s41467-024-50602-8)
Supplement: Supplementary file 1 — Supplementary Information [file 41467_2024_50602_MOESM1_ESM.pdf]

Supplementary Information

Phenotypic heterogeneity follows a growth-viability tradeoff in response to amino acid identity

Kiyan Shabestary, Cinzia Klemm, Benedict Carling, James Marshall, Juline Savigny, Marko Storch, Rodrigo Ledesma-Amaro

Supplementary Figures

Supplementary Figure 1. Overview of the experimental set-ups used in this study.
Supplementary Figure 2. RPL28 scRNAseq reads grouped by scRNAseq cluster.
Supplementary Figure 3. Effect of cell size on pRPL28 fluorescence.
Supplementary Figure 4. Effect of PBS wash duration on heterogeneity.
Supplementary Figure 5. Time course histograms of pRPL28 intensity and cell size for NLIM-PRO and NLIM-GLN.
Supplementary Figure 6. Heterogeneity for NLIM-PRO vs NLIM-GLN.
Supplementary Figure 7. Subpopulation thresholding based on 4 h NLIM-PRO.
Supplementary Figure 8. Comparison between scRNAseq clusters and subpopulation-based RNAsequencing.
Supplementary Figure 9. Subpopulation RNAseq PCA and KEGG enrichment.
Supplementary Figure 10. Timecourse evolution of sorted subpopulations in NLIM-PRO and NLIM-GLN.
Supplementary Figure 11. Mean pRPL28 intensities and cell size for sorted subpopulations in NLIM-PRO and NLIM-GLN.
Supplementary Figure 12. Heterogeneity is dependent on the nitrogen source quality rather than its quantity.
Supplementary Figure 13. Timelapse microscopy of cells shifted to NLIM-PRO.
Supplementary Figure 14. Cell size histogram for wild-type and other laboratory isolates.
Supplementary Figure 15. pRPL28 fluorescence and cell size heterogeneity in response to nitrogen down-shift in NLIM conditions.
Supplementary Figure 16. pRPL28 fluorescence and cell size heterogeneity in response to nitrogen down-shift in NREP conditions.
Supplementary Figure 17. Comparison between replicates for cell size heterogeneity in response to nitrogen down-shift in NLIM conditions.
Supplementary Figure 18. Comparison between replicates for cell size heterogeneity in response to nitrogen down-shift in NREP conditions.
Supplementary Figure 19. Bimodality scores and maximal growth scores for NREP conditions.
Supplementary Figure 20. NREP growth correlation with previous studies.
Supplementary Figure 21. Growth, bimodality and viability parameters for NLIM versus NREP conditions.
Supplementary Figure 22. Population-wide condition-specific viability rates.
Supplementary Figure 23. Timelapse microscopy of sorted fractions grown on YPD pads.

Supplementary Figure 24. Subpopulation-specific viability in PBS and growth resumption in YPD.
Supplementary Figure 25. Comparison between microscopy and flow cytometry for the TF library in 4h NLIM-PRO.
Supplementary Figure 26. Analysis of the TF library.
Supplementary Figure 27. Sensor output over time with Rmixmod clustering for NLIM-PRO.
Supplementary Figure 28. ATP sensor output for all NLIM conditions.

Supplementary Notes

Supplementary Note 1. High and low subpopulations are isogenic.
Supplementary Note 2. Low subpopulation is quiescent.

Supplementary Methods

Supplementary Method 1. Detailed analysis of single-cell RNAseq datasets.

Supplementary Data

Supplementary Data 1. DESeq2 output for scRNAseq data analysis. (xlsx)
Supplementary Data 2. Differentially expressed genes in subpopulation RNAseq. (xlsx)
Supplementary Data 3. Strains used in this study and appearances in figures. (xlsx)
Supplementary Data 4. TF localization ranking. (xlsx)
Supplementary Data 5. Amino acid detection from supernatants of cells exposed to a 8 hours down-shift. (xlsx)

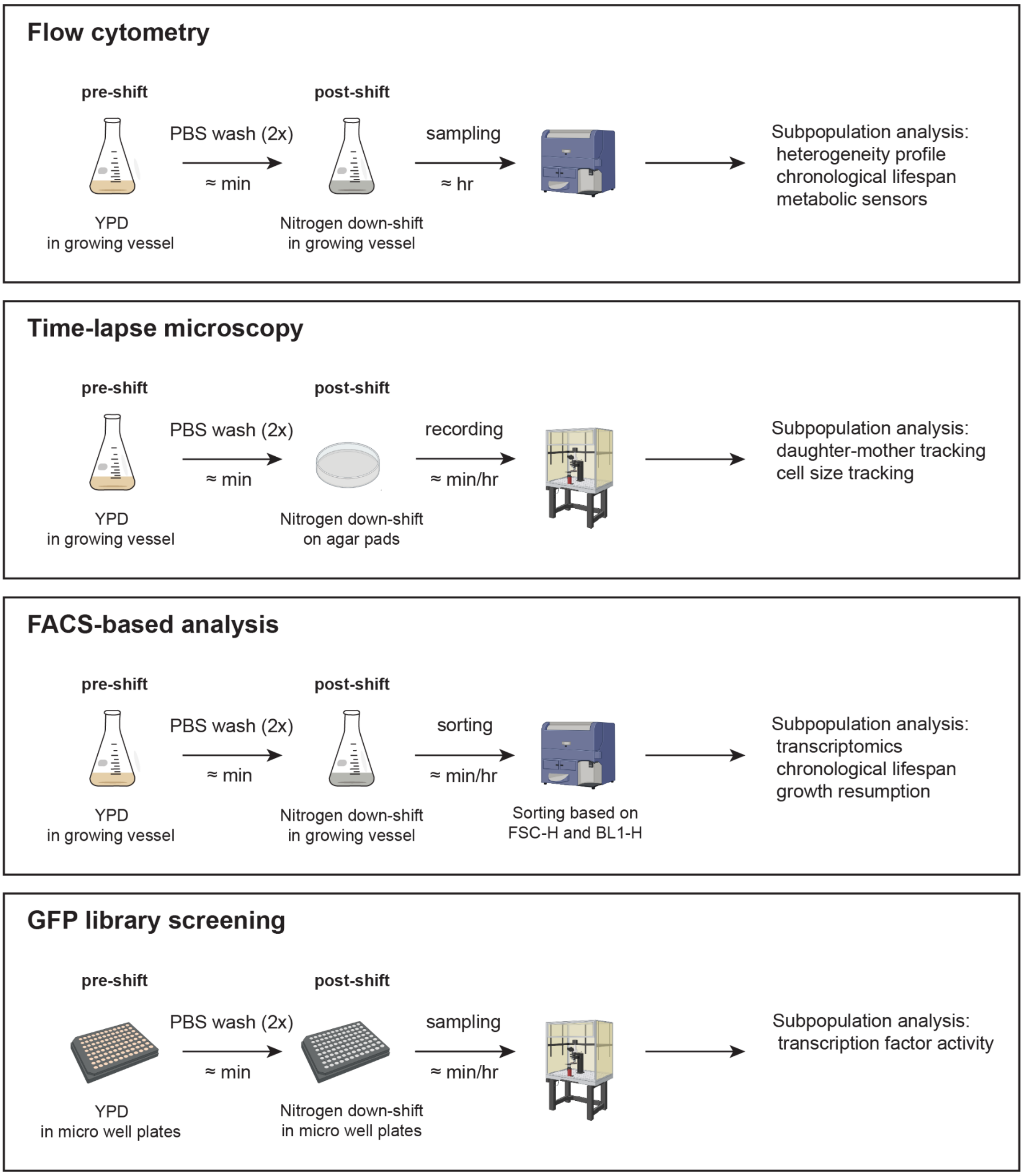

Supplementary Figure 1. Overview of the experimental single-cell methods used in this study. Multi-omics interrogation of cellular differentiation was performed using flow cytometry and microscopy. In short, flow cytometry was used for rapid monitoring of cellular differentiation using cell size (FSC-H), subpopulation marker (BL1-H) as well as chronological lifespan. Time-lapse microscopy on agar pads allowed single cell tracking to unravel population dynamics over time using neural network-based segmentation tools. FACS was used for subpopulation separation and further analysis (subpopulation transcriptomics, chronological life span and growth resumption in nitrogen-limited and rich media). Finally, high-throughput microscopy was performed on a prototrophic GFP library to investigate transcription factor response (96 transcription factors). Flow cytometer, microscope and plates schematics were made with BioRender.com released under a CC-BY-NC-ND 4.0 International license.

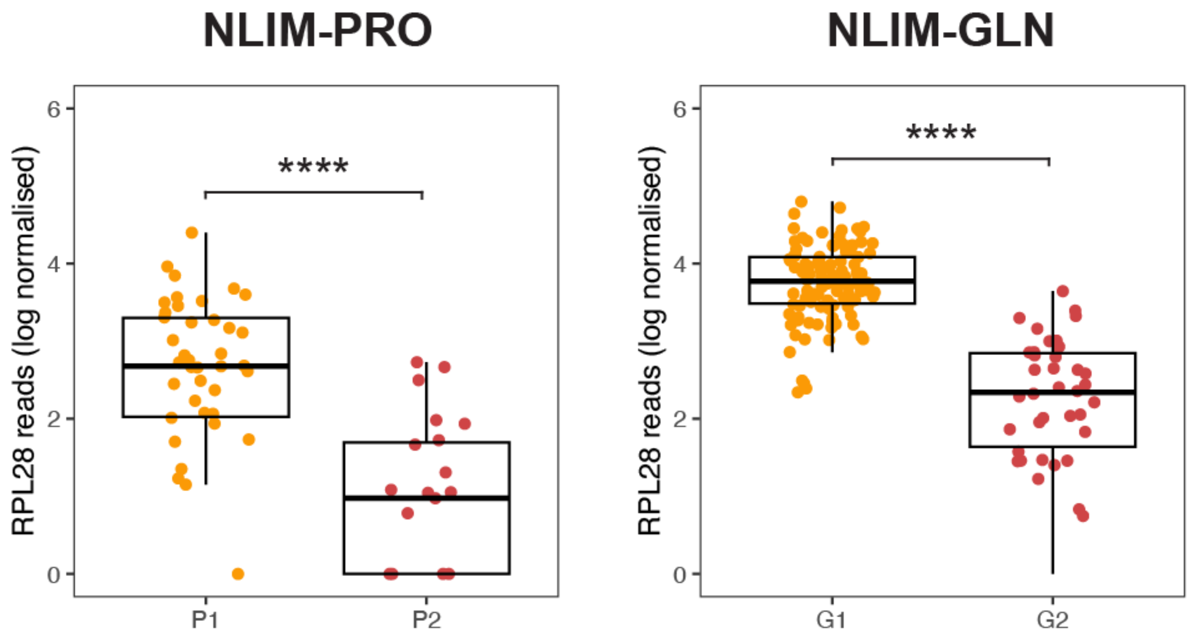

Supplementary Figure 2. RPL28 scRNAseq reads grouped by scRNAseq cluster. Reads from single-cell RNA sequencing[1] were log-normalised and grouped according to UMAP-based clustering (Fig. 1c). Only WT phenotypes were considered in the analysis. Significance scores denotes p-value of unpaired two-sided t-test with $p < 0.00005$ (****).

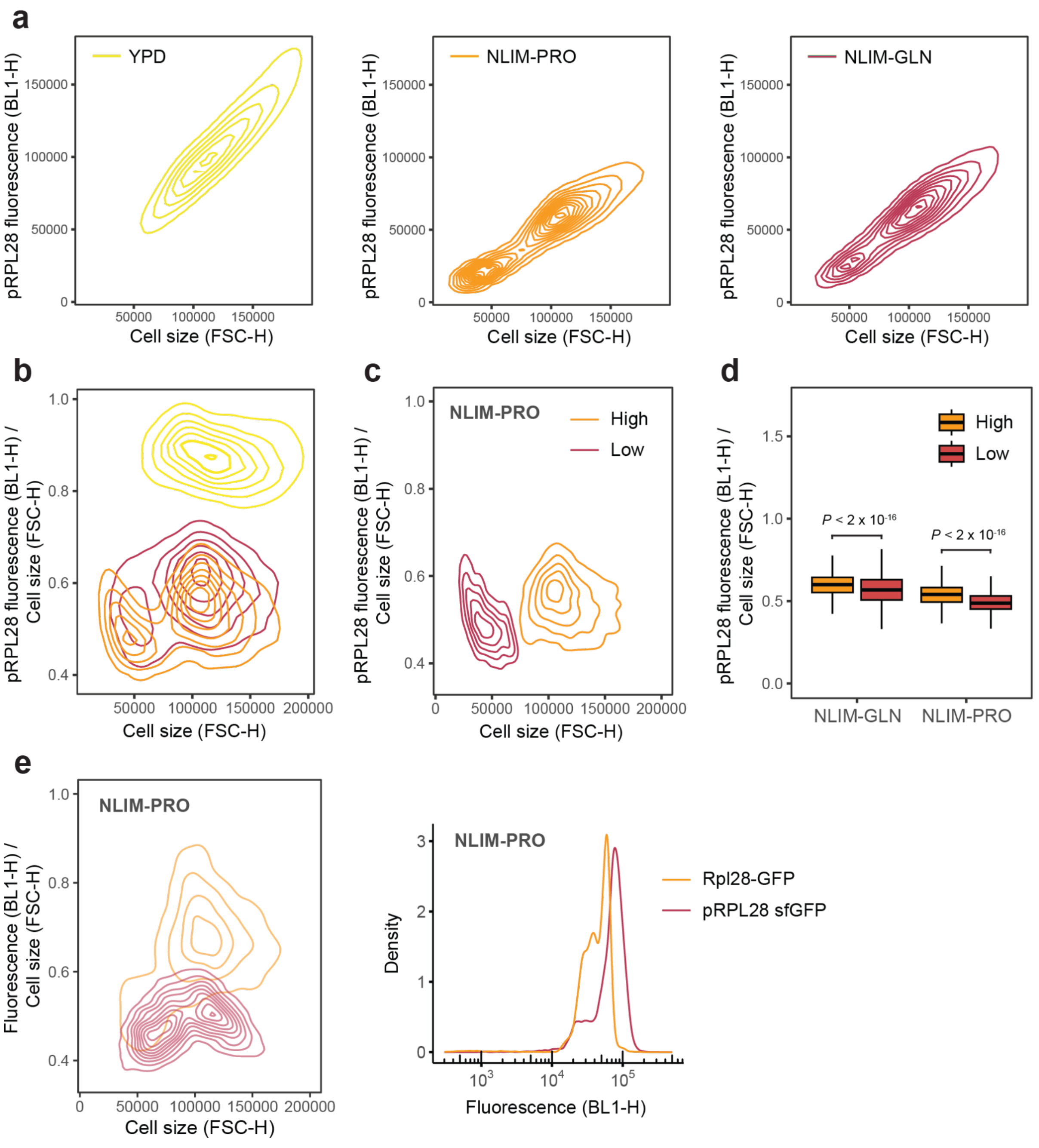

Supplementary Figure 3. Effect of cell size on pRPL28 fluorescence and comparison with Rpl28-tagged strain. (a) Contour plot of FSC-H/BL1-H for YPD, NLIM-PRO and NLIM-GLN. (b) Contour plot where fluorescence has been normalised by cell size (FSC-H). (c) Contour plot after high and low subpopulation have been clustered (Methods). (d) Box plot showing significant differences in pRPL28 intensity per cell size (BL1-H/FSC-H) for NLIM-PRO and NLIM-GLN. (e) Signal comparison between pRPL28 driven sfGFP and Rpl28-tagged GFP fluorescence. Data is 4 h post-shift.

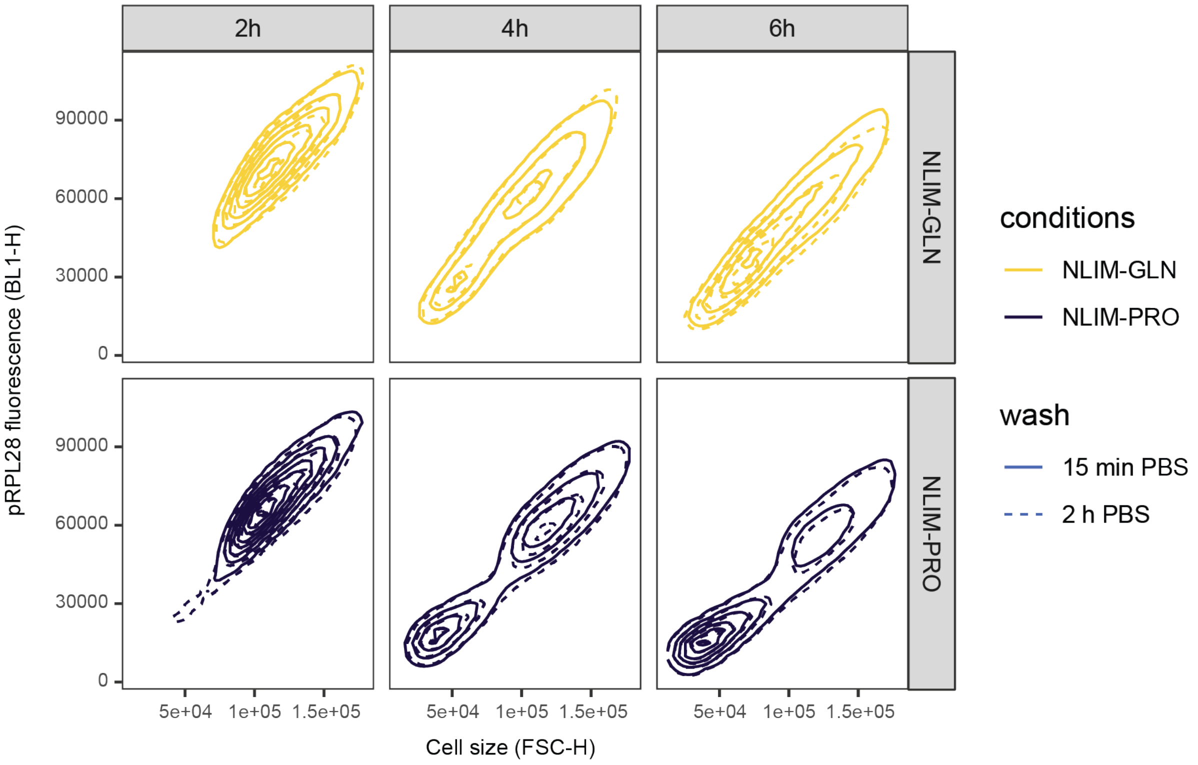

Supplementary Figure 4. Effect of PBS wash duration on heterogeneity. Sold line (15 min wash) represents the default washing time throughout this paper. Dashed line (2 h wash) represents cells that were washed (as in 15 min wash) but left in PBS for 2 more hours.

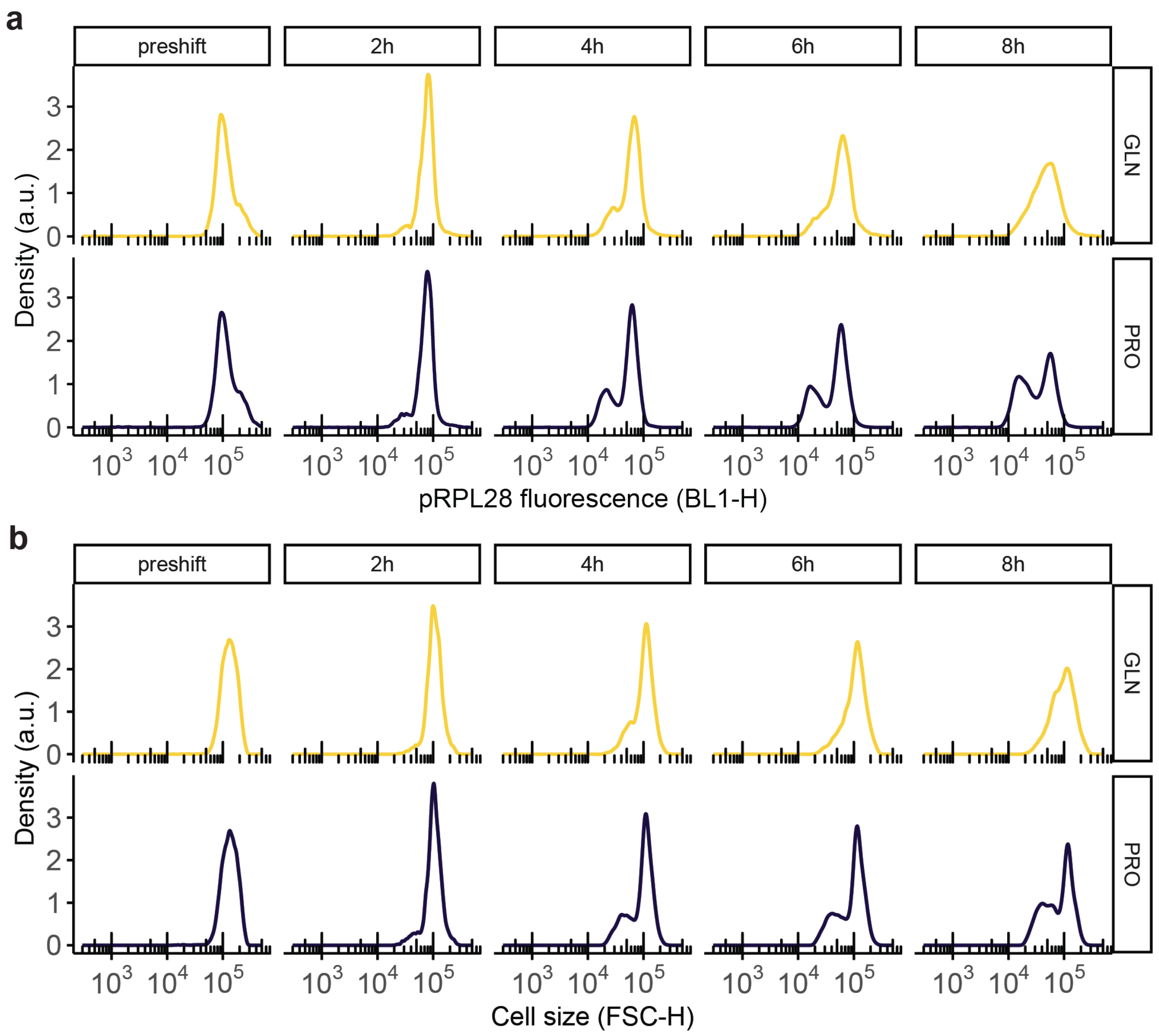

Supplementary Figure 5. Time course histograms of pRPL28 intensity and cell size for NLIM-PRO and NLIM-GLN. Histograms indicate pRPL28 fluorescence (a) and cell size (b). Preshift indicates distributions in YPD prior to the shift.

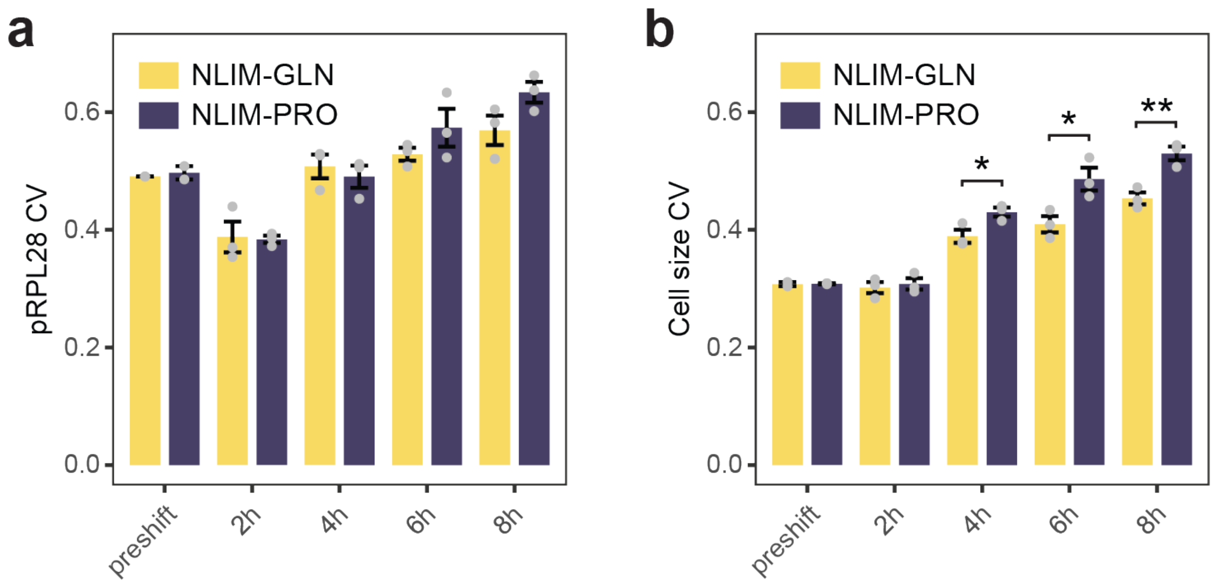

Supplementary Figure 6. Heterogeneity for NLIM-PRO vs NLIM-GLN. Coefficient of variation (CV) for (a) pRPL28 intensity and (b) cell size. The coefficient of variation CV is defined as the population standard deviation over the population mean. Significance scores denotes conditions where all replicates had a p-value (one-tailed t-test) of at least $p < 0.005$ (*) or $p < 0.005$ (**).

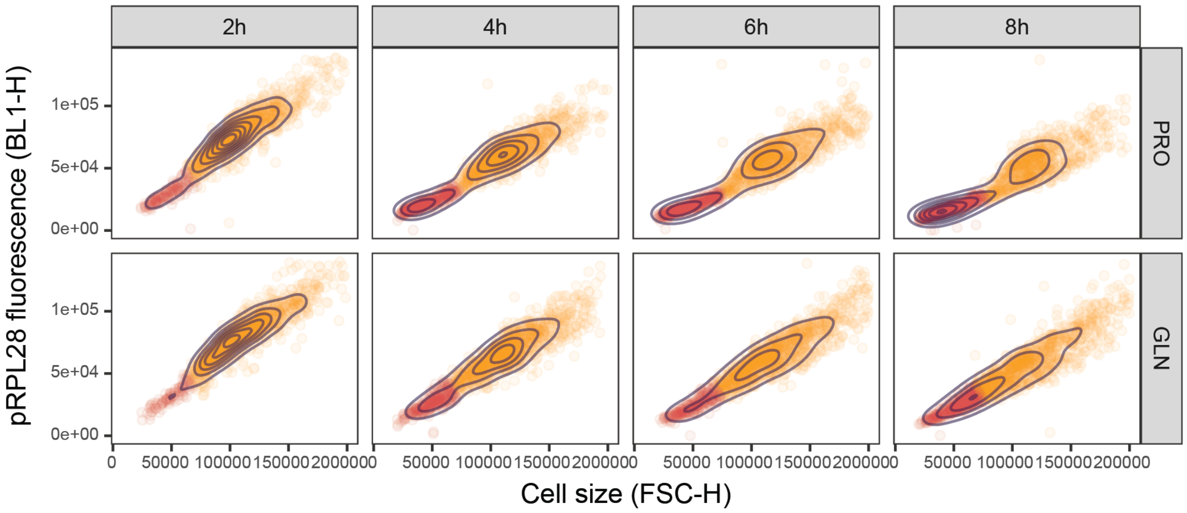

Supplementary Figure 7. Subpopulation thresholding based on 4 h NLIM-PRO. Grey contours indicate the whole population density. Individual points stand for individual cells. In red and orange, cells assigned to low and high subpopulation, respectively. Assignment was based on previous clustering using expectation maximisation for NLIM-PRO 4h.

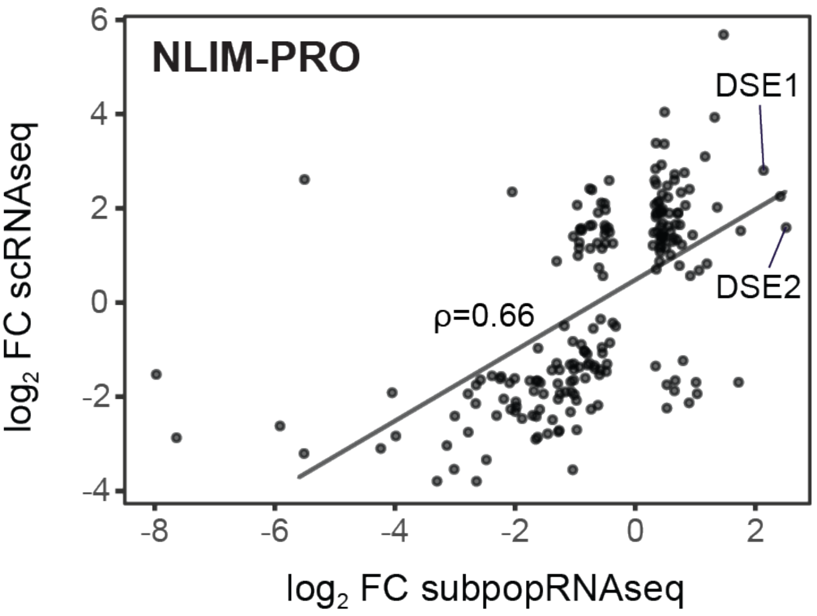

Supplementary Figure 8. Comparison between scRNAseq clusters and subpopulation-based RNAsequencing. Correlation between scRNAseq (from ref.[1]; 4 h postshift) and subpopRNAseq (this study; 30 min postshift). Comparison was performed for NLIM-PRO on differentially expressed genes (p-adj <= 0.05) between low versus high subpopulations (subpopRNAseq) and P2 versus P1 clusters (scRNAseq).

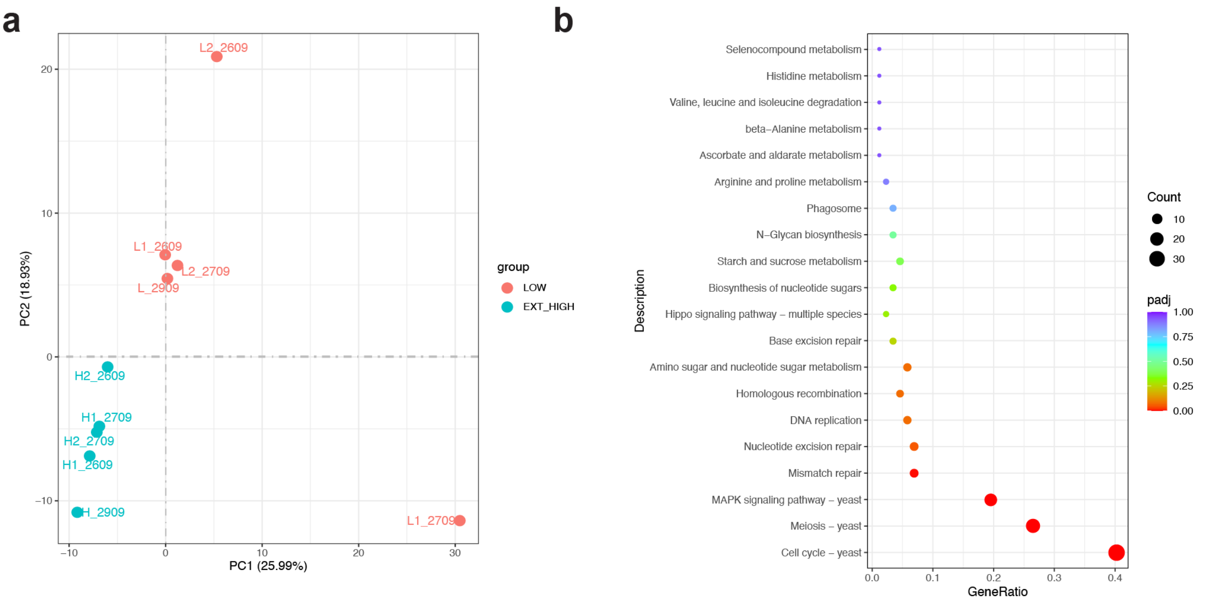

Supplementary Figure 9. Subpopulation RNAseq PCA and KEGG enrichment. (a) Principal component analysis (PCA) was used to remove outliers such as samples L2_2609 and L1_2709. **(b)** KEGG enrichment (bi-directional) of genes that were significantly (p_{adj} < 0.05) differentially regulated between high and low subpopulations.

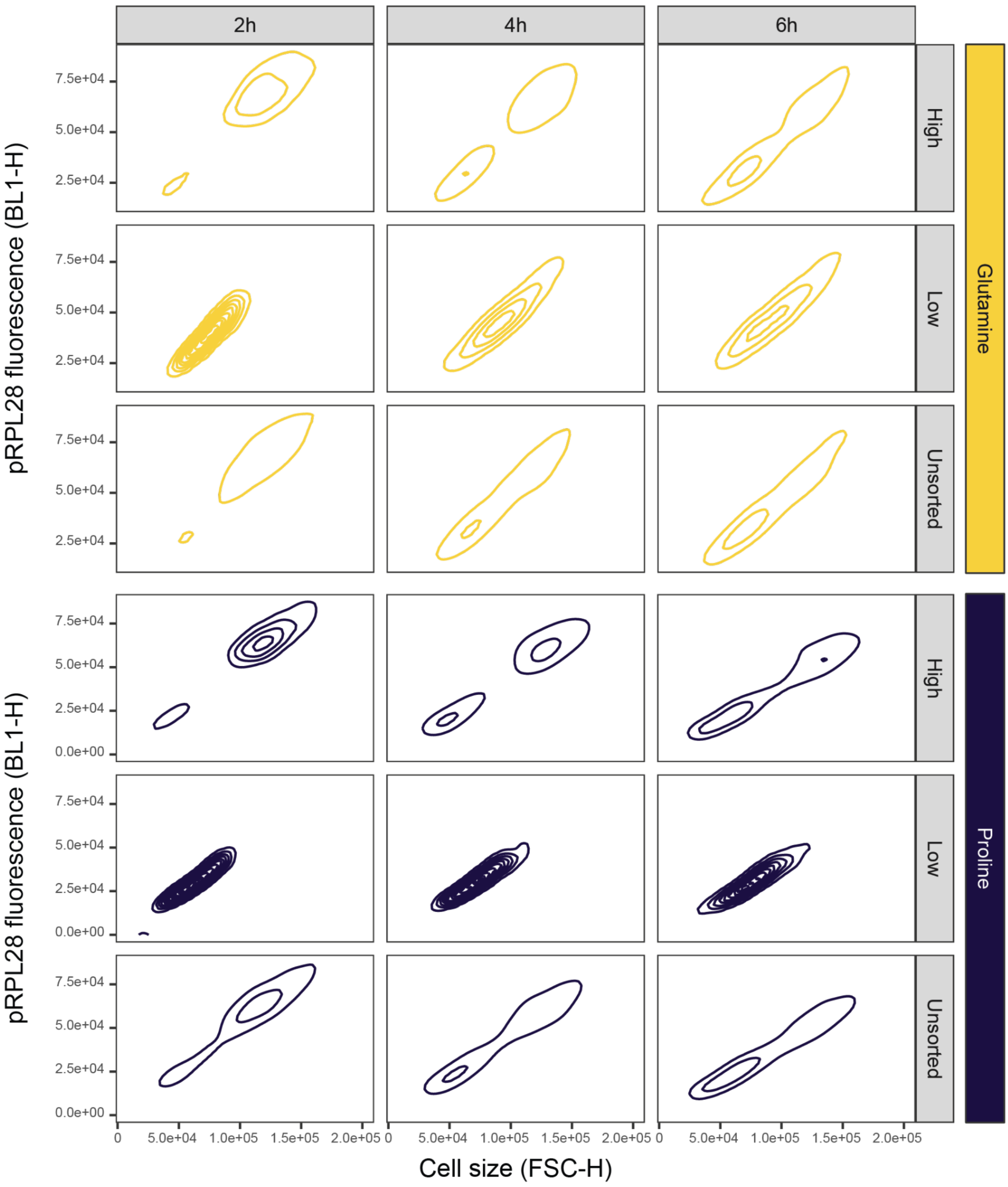

Supplementary Figure 10. Timecourse evolution of sorted subpopulations in NLIM-PRO and NLIM-GLN. Subpopulations were grown for 2 h in NLIM-PRO or NLIM-GLN were sorted and put back in their respective media. Scatter plot of pRPL28 intensity and cell size shows the dynamics of the subpopulation.

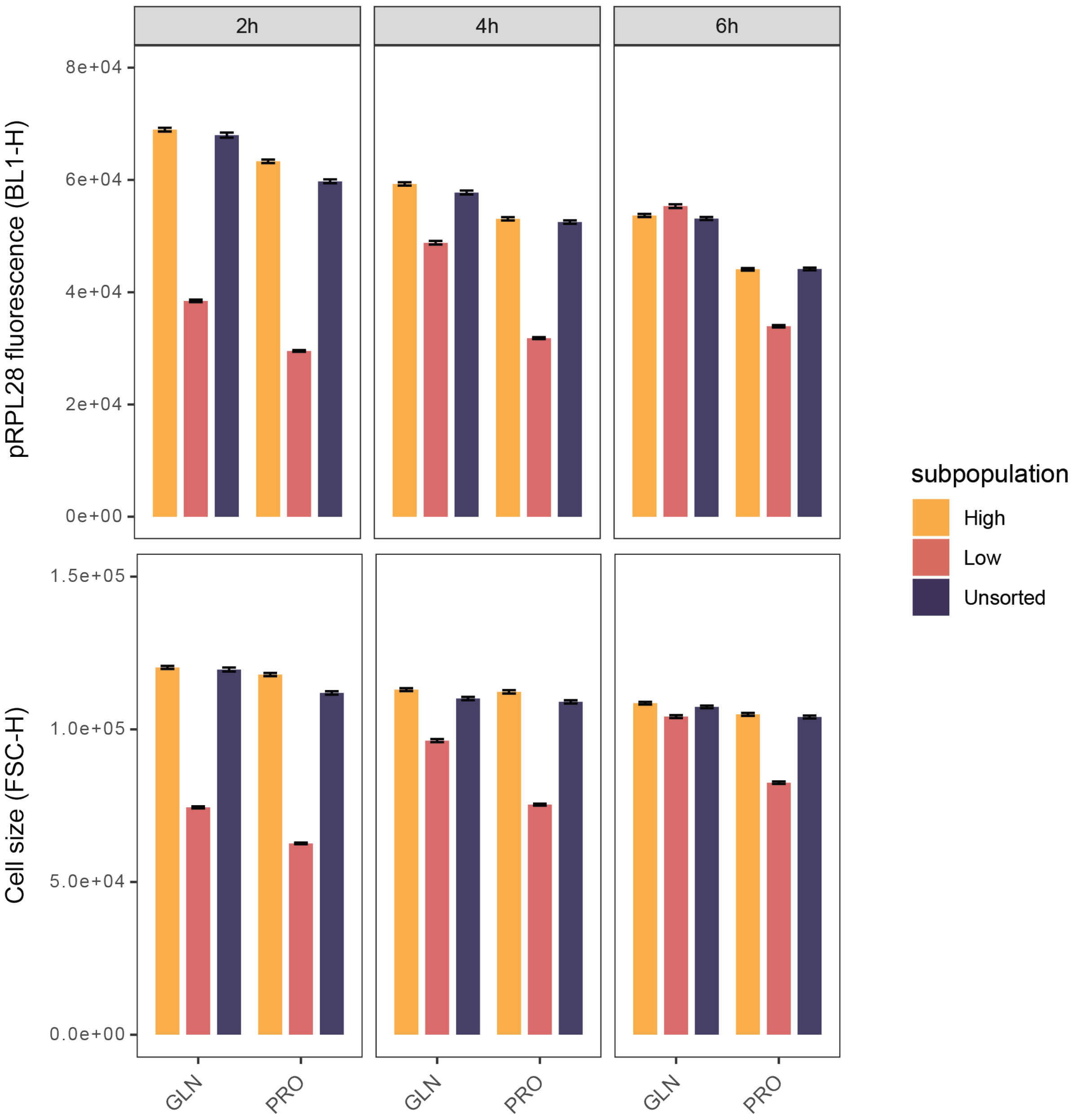

Supplementary Figure 11. Mean pRPL28 intensities and cell size for sorted subpopulations in NLIM-PRO and NLIM-GLN. Subpopulations were grown for 2 h in NLIM-PRO or NLIM-GLN were sorted and put back in their respective media.

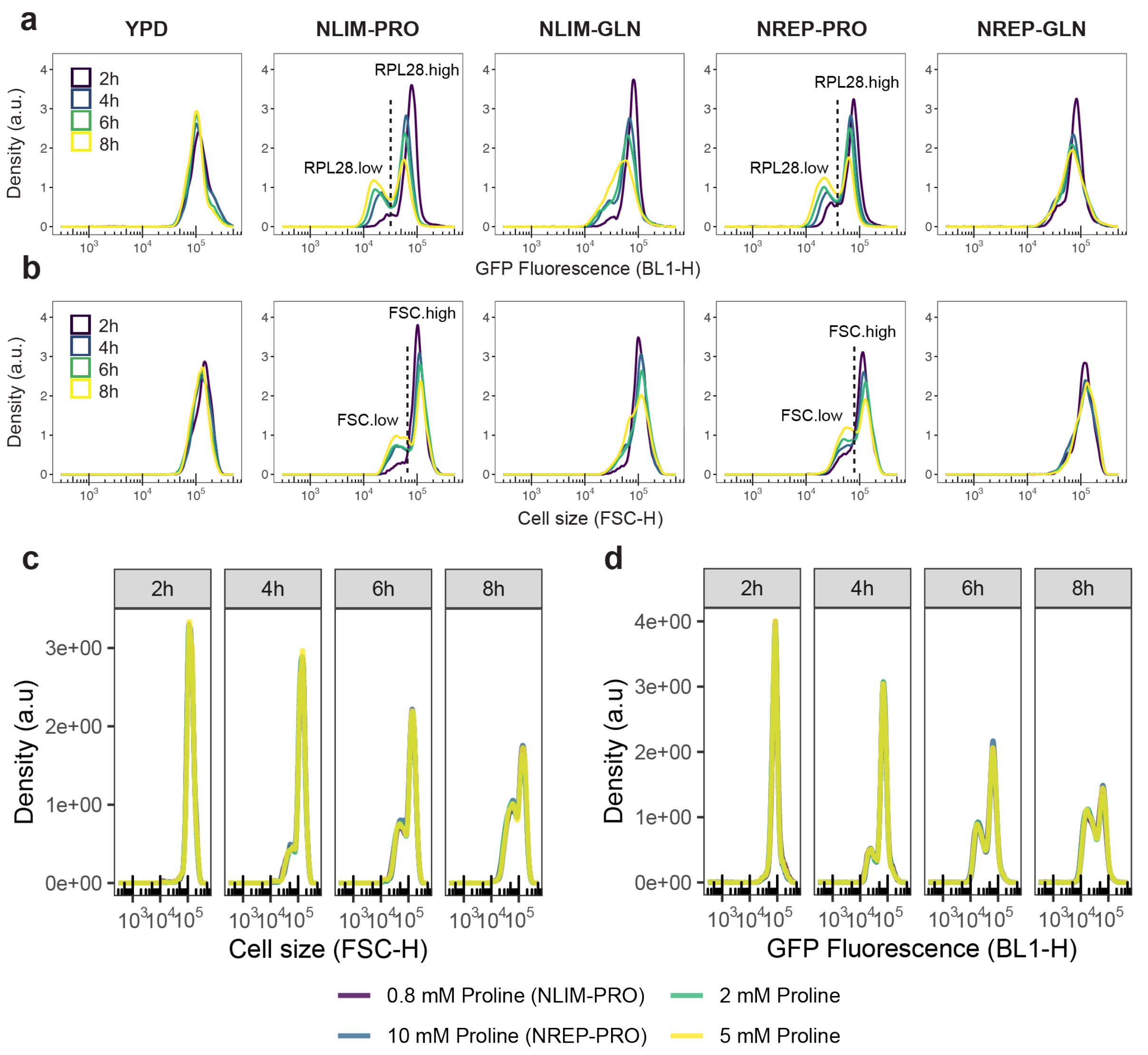

Supplementary Figure 12. Heterogeneity is dependent on the nitrogen source quality rather than its quantity. (a) pRPL28 and **(b)** cell size heterogeneity are compared to the NLIM condition. Different proline concentrations were tested to show the conserved heterogeneity with respect to varying conditions for **(c)** cell size **(d)** pRPL28 intensity.

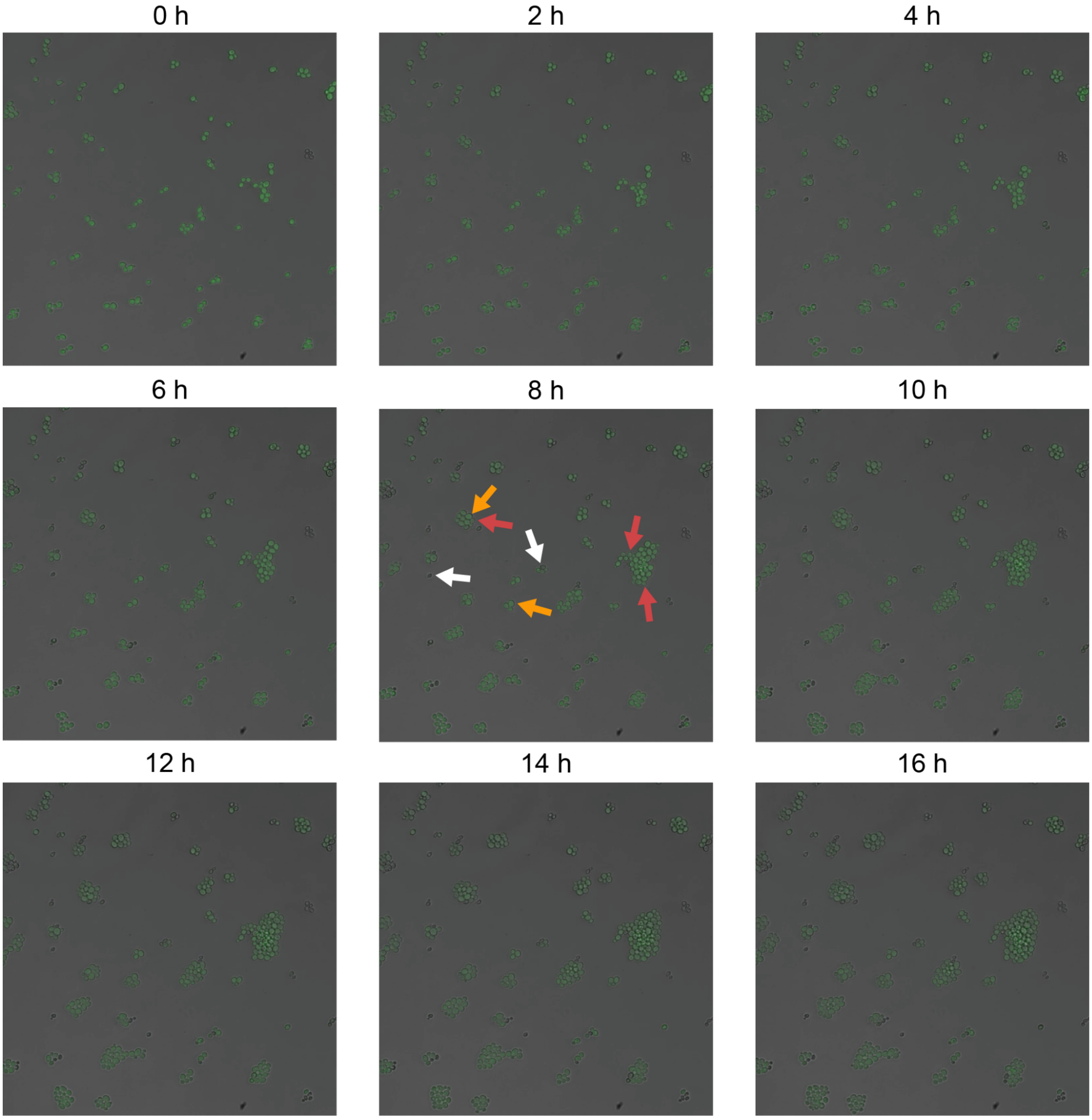

Supplementary Figure 13. Timelapse microscopy of cells shifted to NLIM-PRO. Cells were grown in rich media, washed in PBS twice and applied to an agar pad made of NLIM-PRO. The agar pad was then placed upside down on a microscopy slide within an imaging dish. Brightfield and GFP fluorescence were recorded over time with 20X magnification. White arrows indicate cells that have lost their fluorescence, red arrows cells seemingly from the low subpopulations and orange from the high subpopulations.

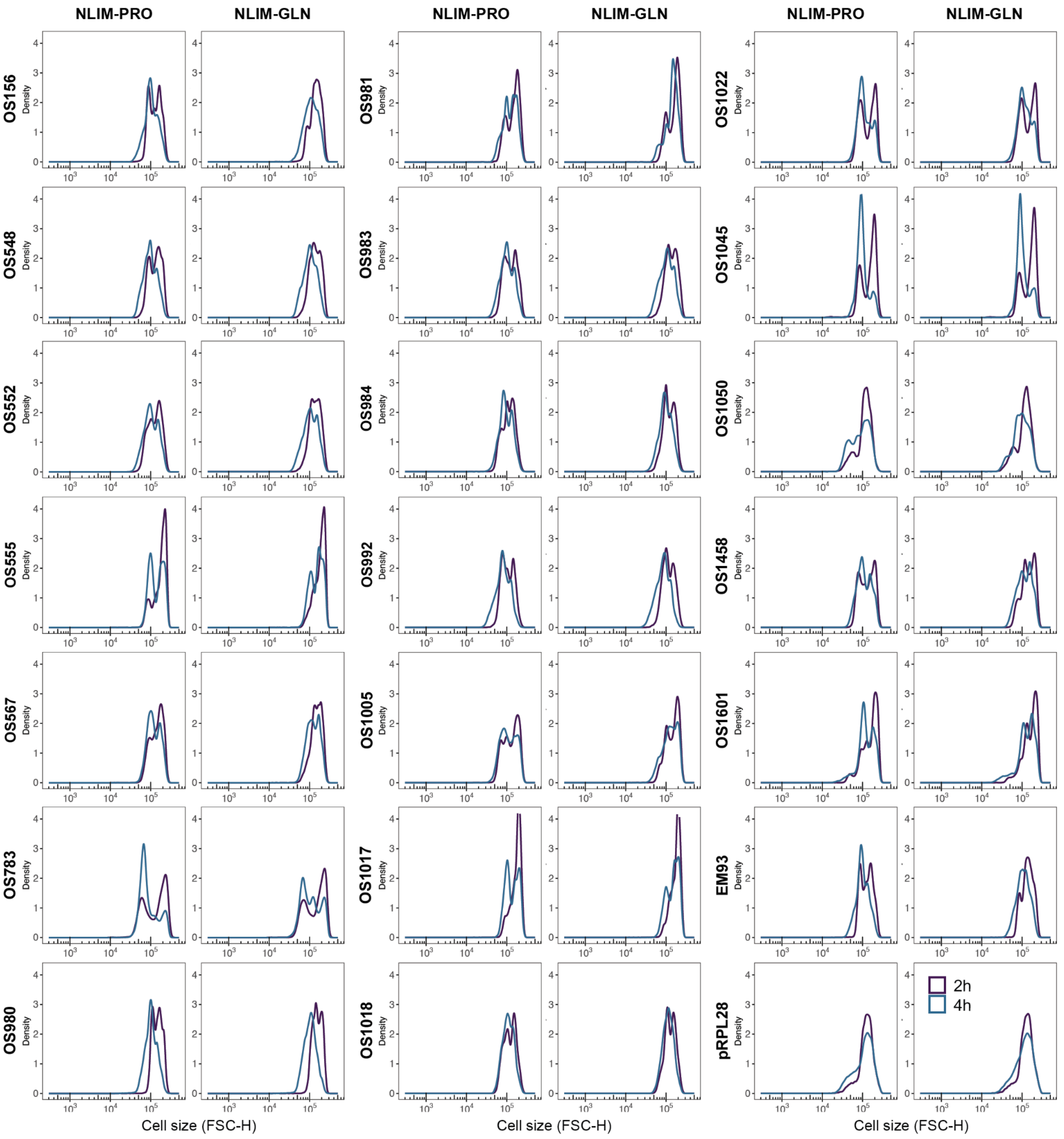

Supplementary Figure 14. Cell size histogram for wild-type and other laboratory isolates. Cells were shifted to NLIM-PRO and NLIM-GLN. For strain reference and origin, the reader is referred to Supplementary Data 3. Flow cytometry data are shown for one of two biological replicates tested. Strain pRPL28 is the strain used throughout this study.

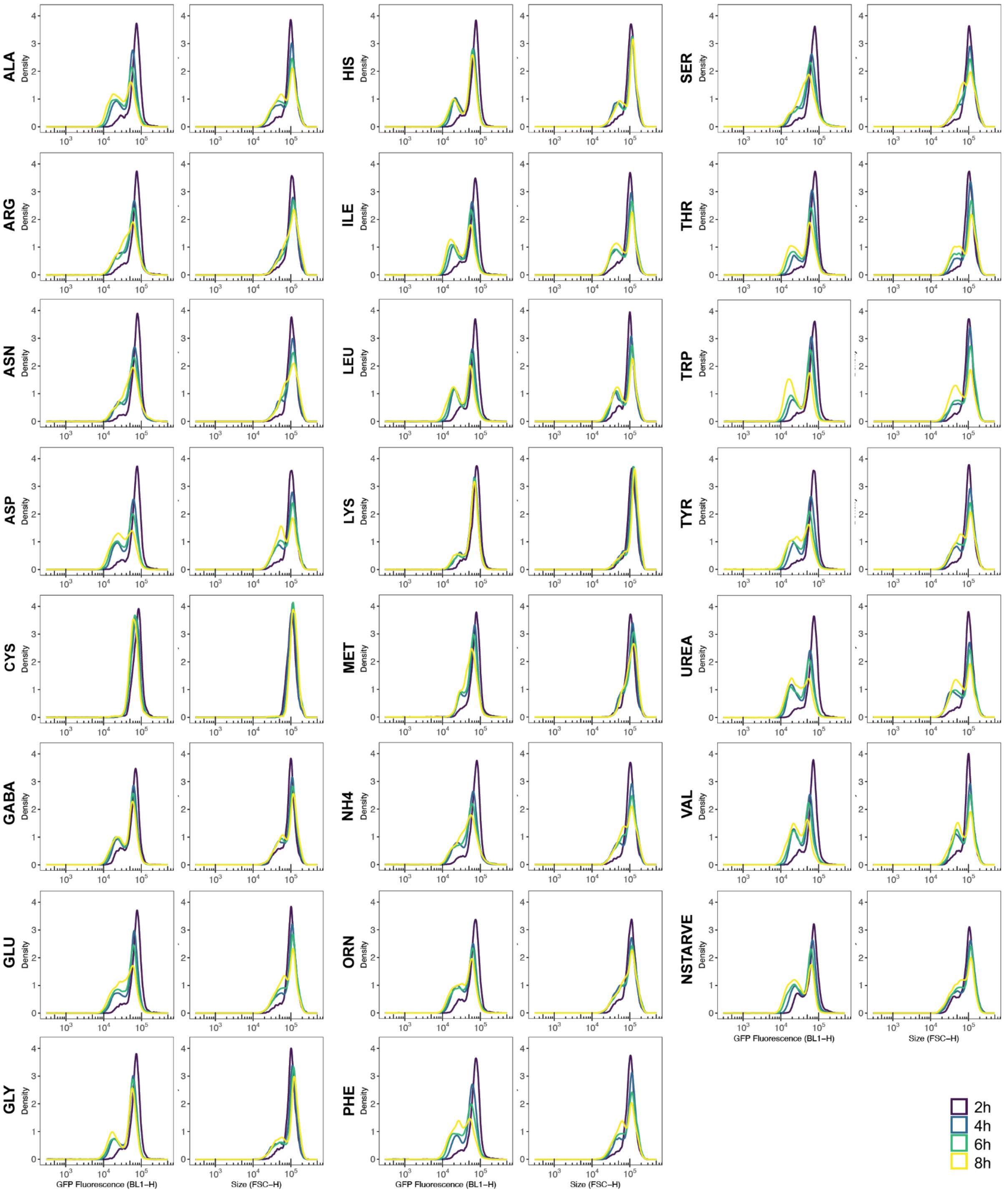

Supplementary Figure 15. pRPL28 fluorescence and cell size heterogeneity in response to different nitrogen down-shift in NLIM. Data is for one representative out of three experiments.

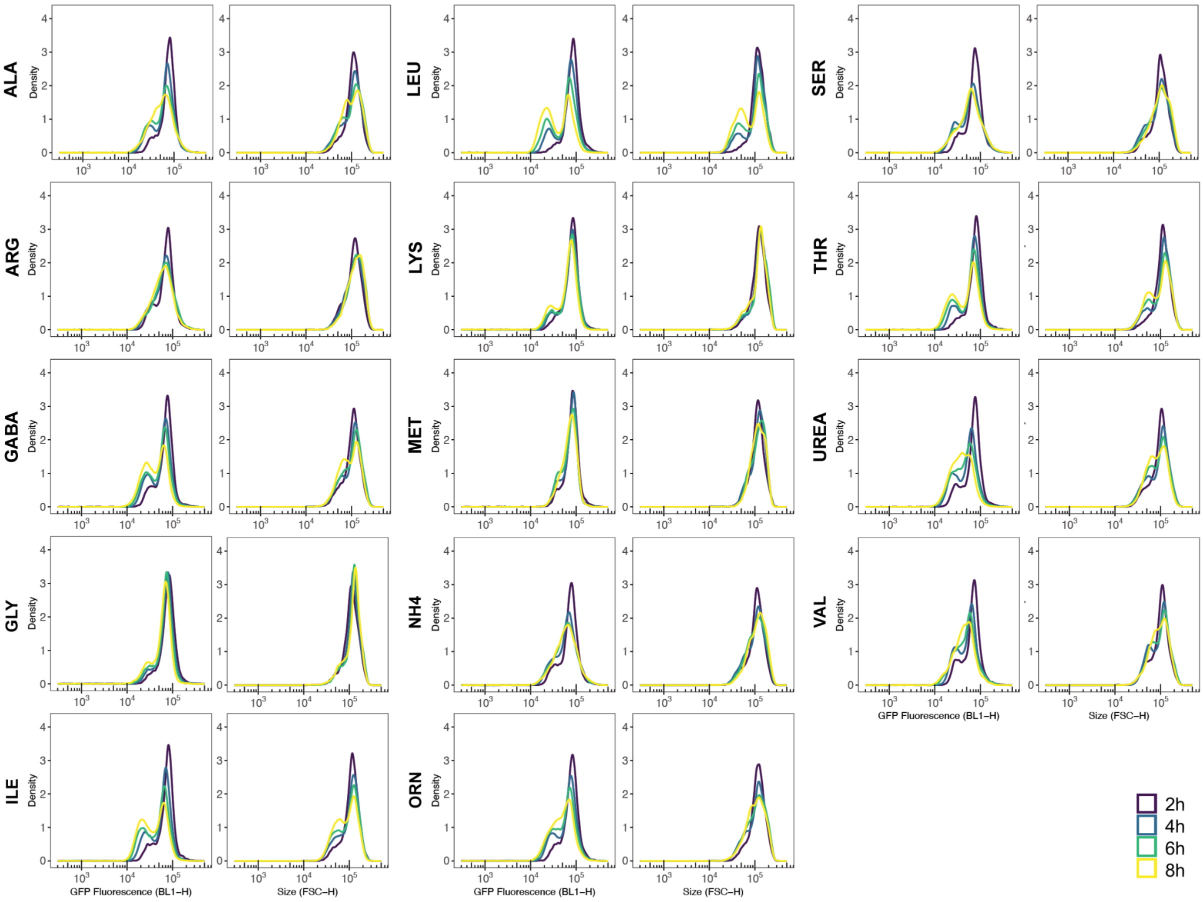

Supplementary Figure 16. pRPL28 fluorescence and cell size heterogeneity in response to different nitrogen down-shift in NREP. Data is for one representative out of three experiments.

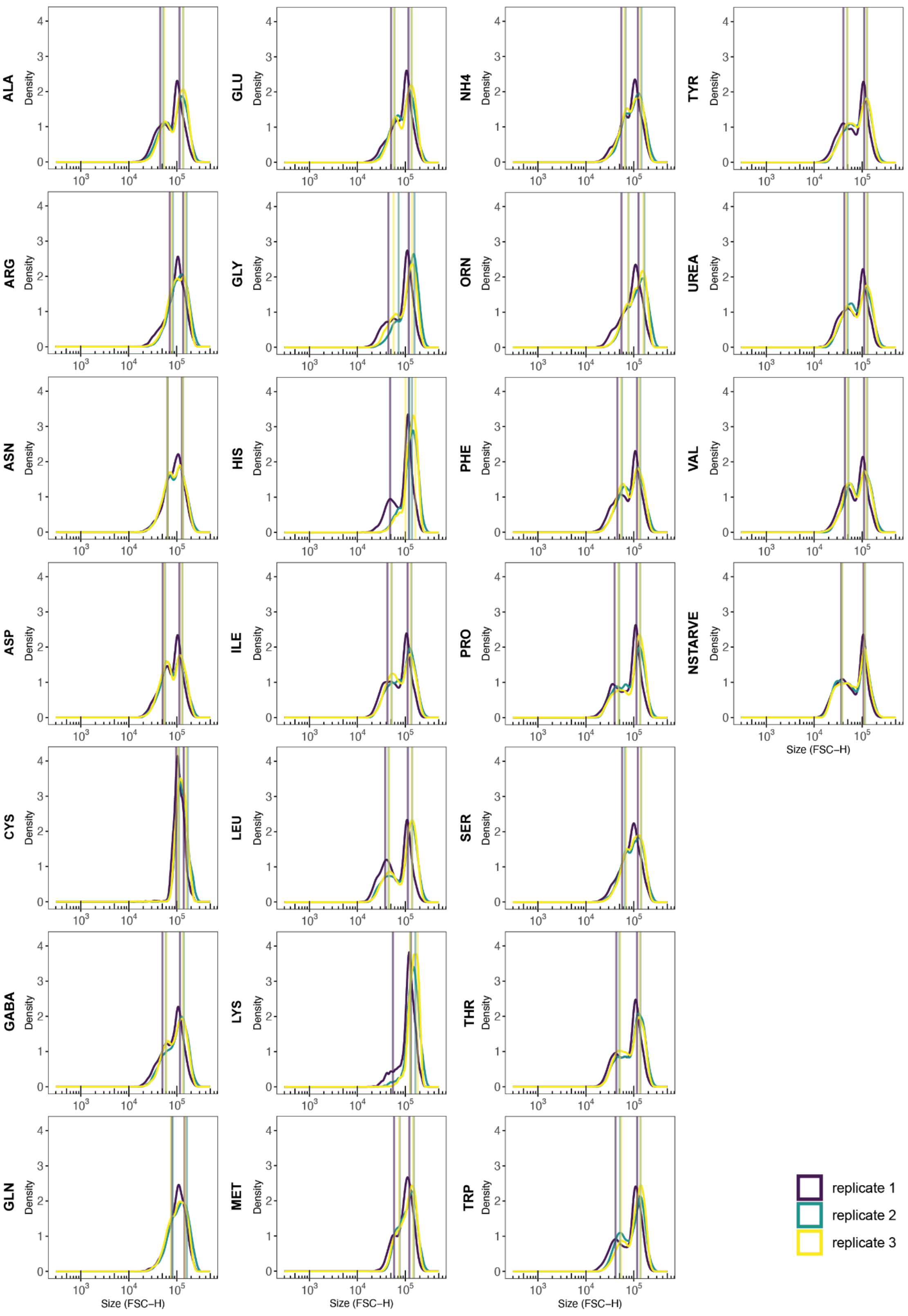

Supplementary Figure 17. Comparison between replicates for cell size heterogeneity in response to nitrogen down-shift in NLIM. Cells were exposed to a 6 h down-shift for replicates performed on different days. Lines represent predicted mean of the subpopulation using the flexmix package (Methods). Replicates (n = 3) were from flow cytometry runs performed on different days.

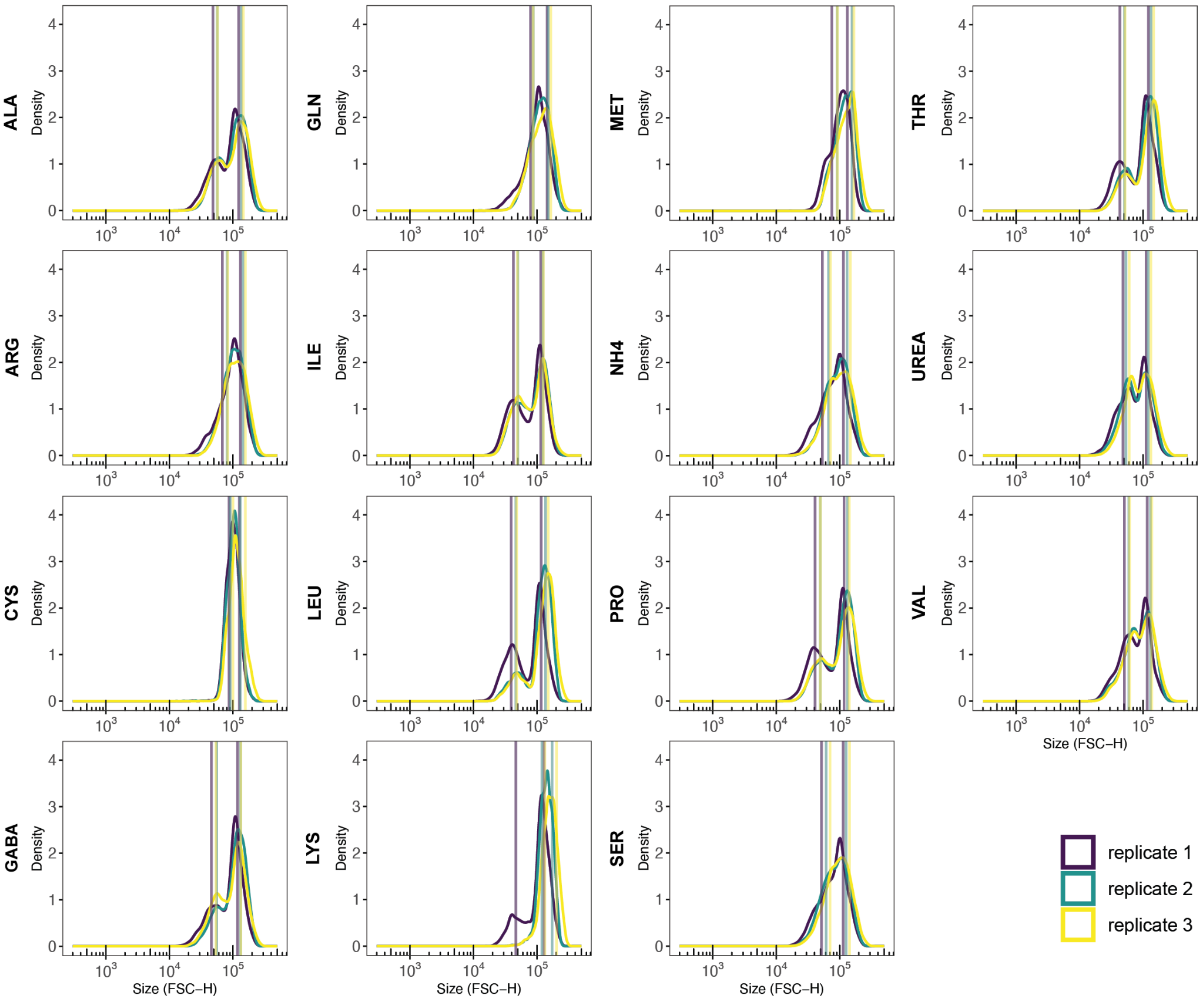

Supplementary Figure 18. Comparison between replicates for cell size heterogeneity in response to nitrogen down-shift in NREP. Cells were exposed to a 6 h down-shift for replicates performed on different days. Lines represent predicted mean of the subpopulation using the flexmix package (Methods). Replicates (n = 3) were from flow cytometry runs performed on different days.

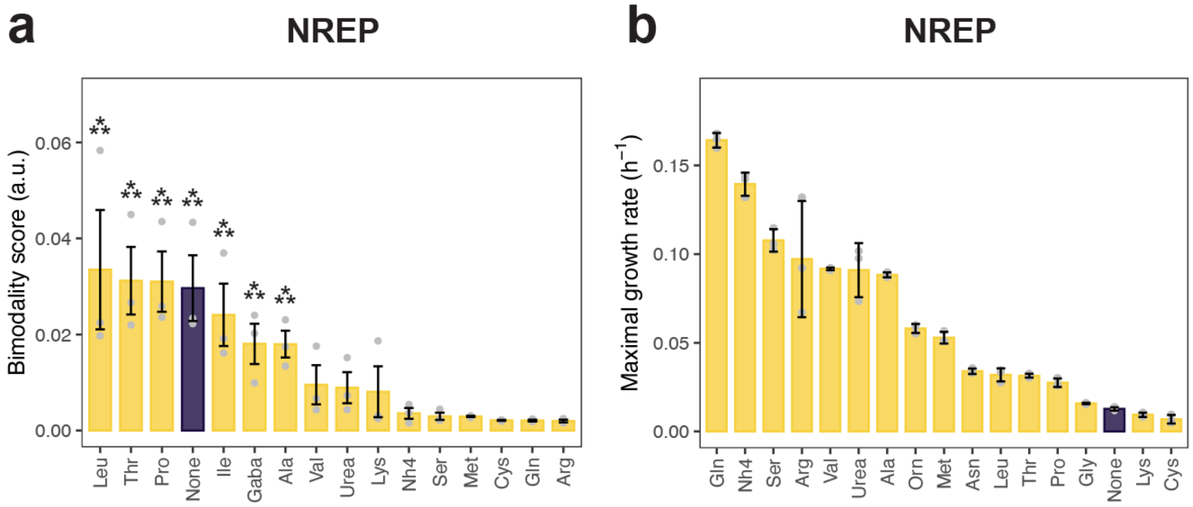

Supplementary Figure 19. Bimodality scores and maximal growth scores for NREP conditions. (a) Bimodality scores (Hartigan's diptest) and **(b)** maximal growth rates across all NREP conditions. Bar plot represents the mean ± SEM of (a) flow cytometry experiments for cells exposed to a 6 h down-shift performed on different days (n = 3) and (b) growth curves of biological replicates (n = 3). Purple bars represent the NSTARVE condition where no nitrogen is present. Significance scores denotes conditions where all replicates had a p-value (Hartigan's diptest) of at least p < 0.05 (*), p < 0.005 (**) or p < 0.0005 (***). Bimodality scores are shown as arbitrary units (abbreviated a.u.).

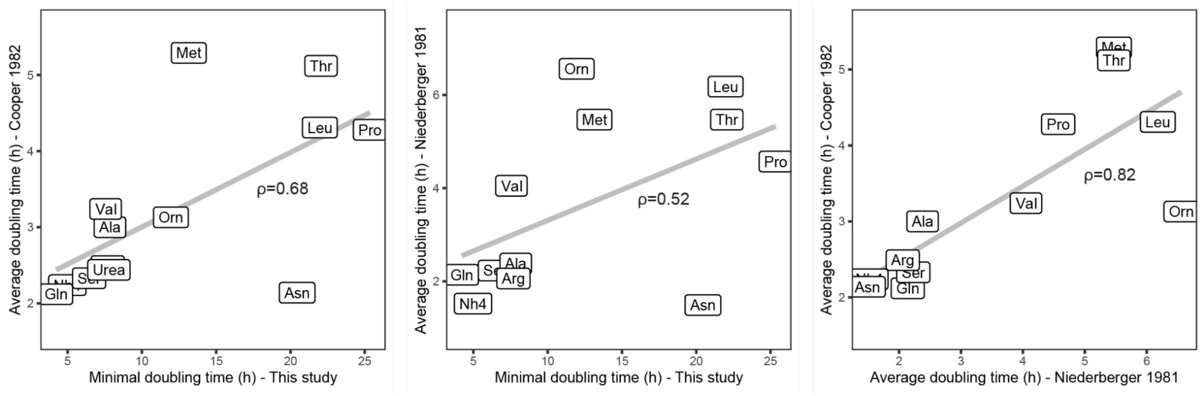

Supplementary Figure 20. NREP growth correlation with previous studies. Maximal growth rates calculated in this study and converted in minimal doubling times were compared with previous datasets summarised and reviewed in Ljungdahl et al.[2]. Regression line is indicated in grey. Scores denote Spearman rank correlations.

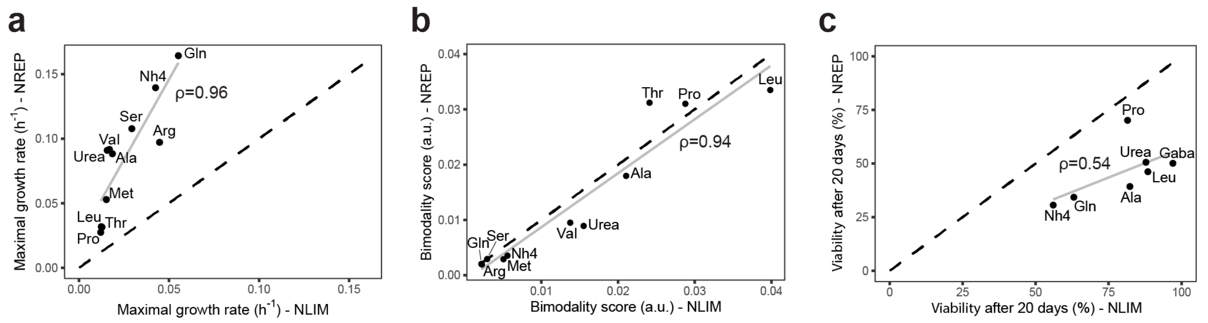

Supplementary Figure 21. Growth, bimodality and viability parameters for NLIM versus NREP conditions. (a) Maximal growth rate, **(b)** bimodality and **(c)** viability score for NLIM versus NREP conditions of cells exposed to a 8h downshift. Regression line is indicated in grey with spearman rank correlations. Dashed line represents the identity line between NLIM and NREP conditions.

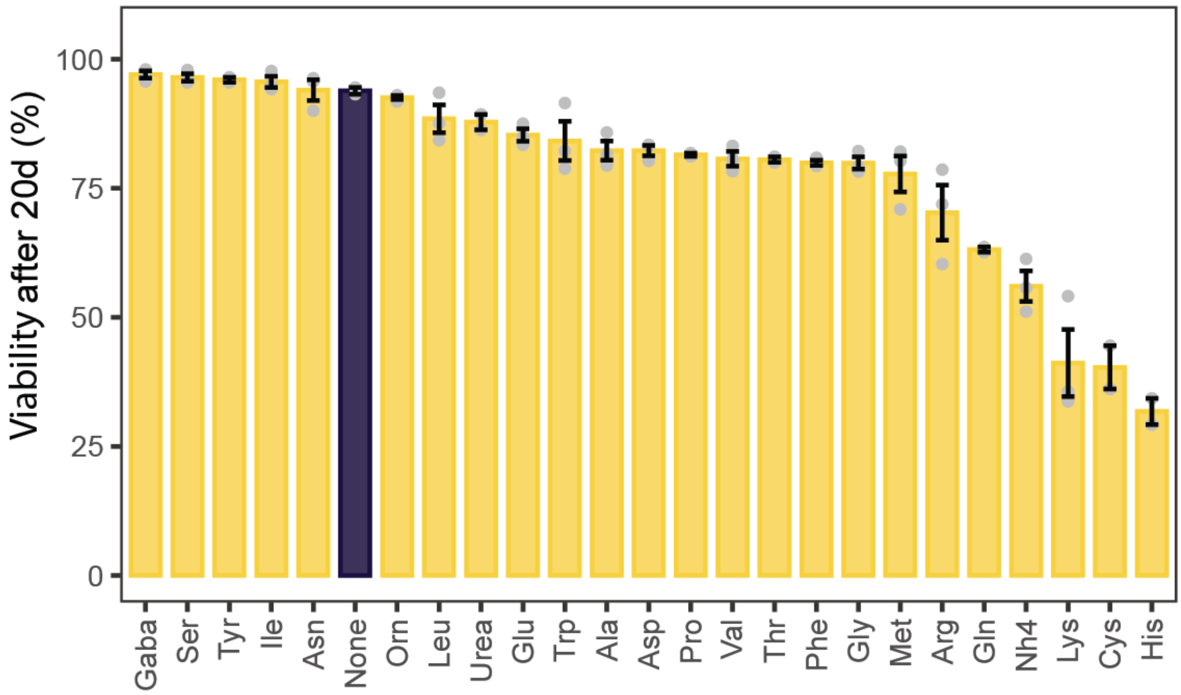

Supplementary Figure 22. Population-wide condition-specific viability rates. Viability rates of cells exposed to a 18 h nitrogen downshift in NLIM (yellow) or NSTARVE (dark purple) and stored 20 days in PBS, measured by flow cytometry and thresholded through propidium iodide staining.

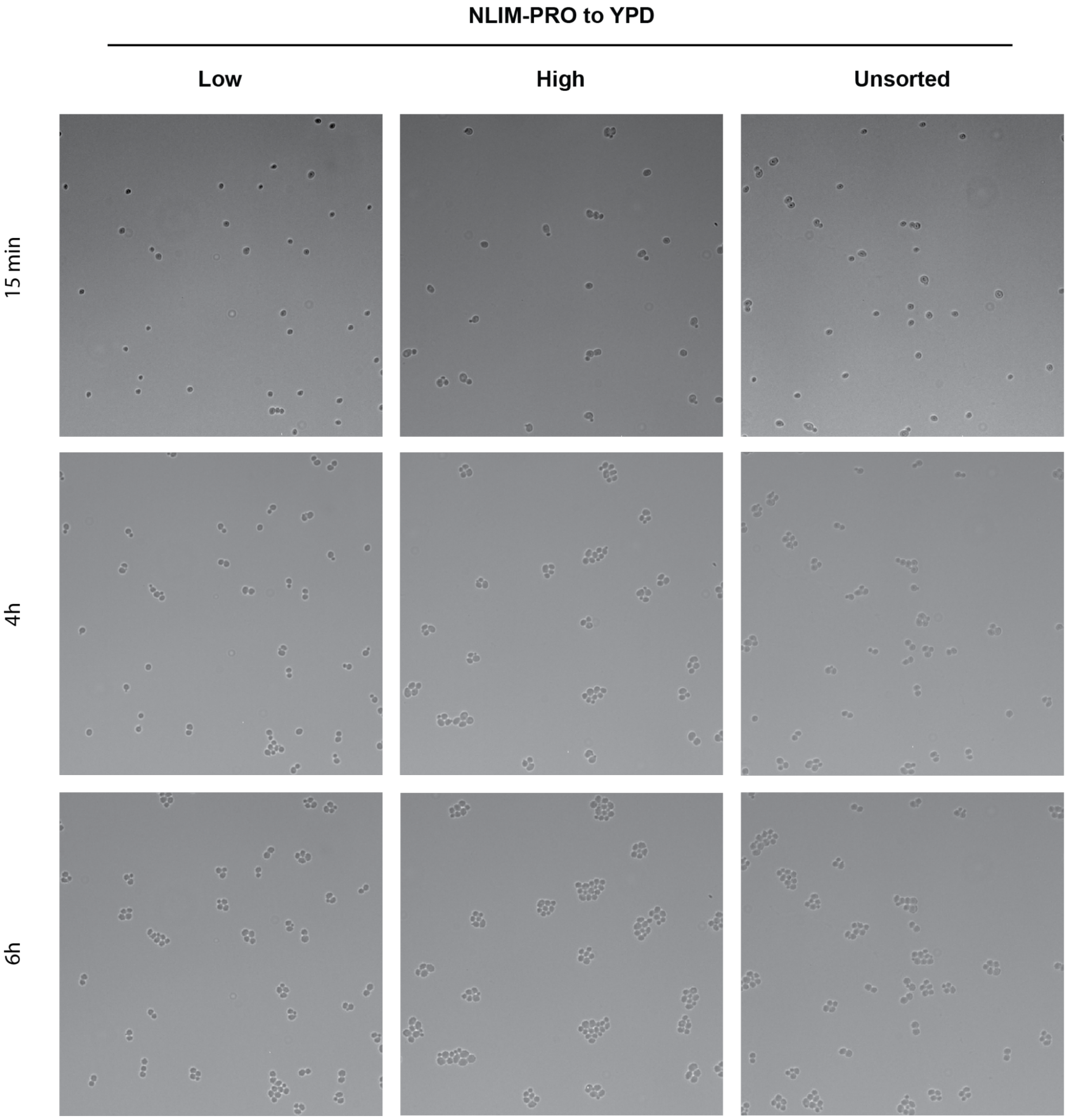

Supplementary Figure 23. Timelapse microscopy of sorted fractions grown on YPD pads. Cells were exposed to NLIM-PRO for 2 h and sorted based on pRPL28 intensity and cell size as previously described. As observed at the bulk subpopulation level, the high subpopulation has a shorter lag phase with higher number of doublings observed when compared to the low subpopulation. Time represents the time spent on agar pads prior to imaging. Magnification is 20X.

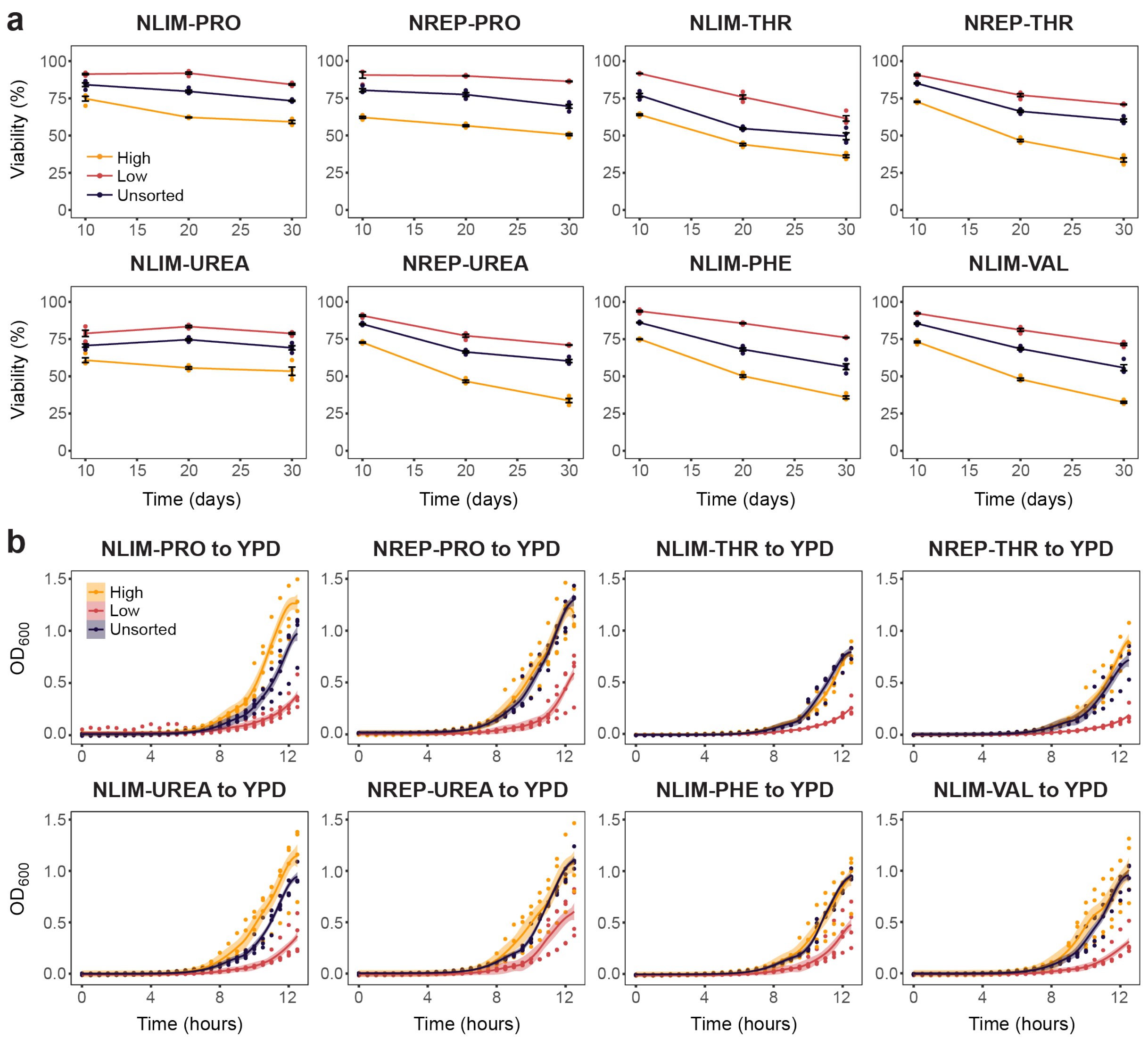

Supplementary Figure 24. Subpopulation-specific viability in PBS and growth resumption in YPD. (a) Viability rates for cells exposed to a 4 h downshift and sorted based on size and pRPL28 intensities using FACS. Yellow and pink samples indicate high and low GFP fractions. Dark purple samples indicate cells passed through FACS but not gated. **(b)** Growth resumption in rich media (YPD) for sorted and unsorted fractions (starting OD_{600} = 0.05). Shaded area represents the 95% confidence interval (n = 4).

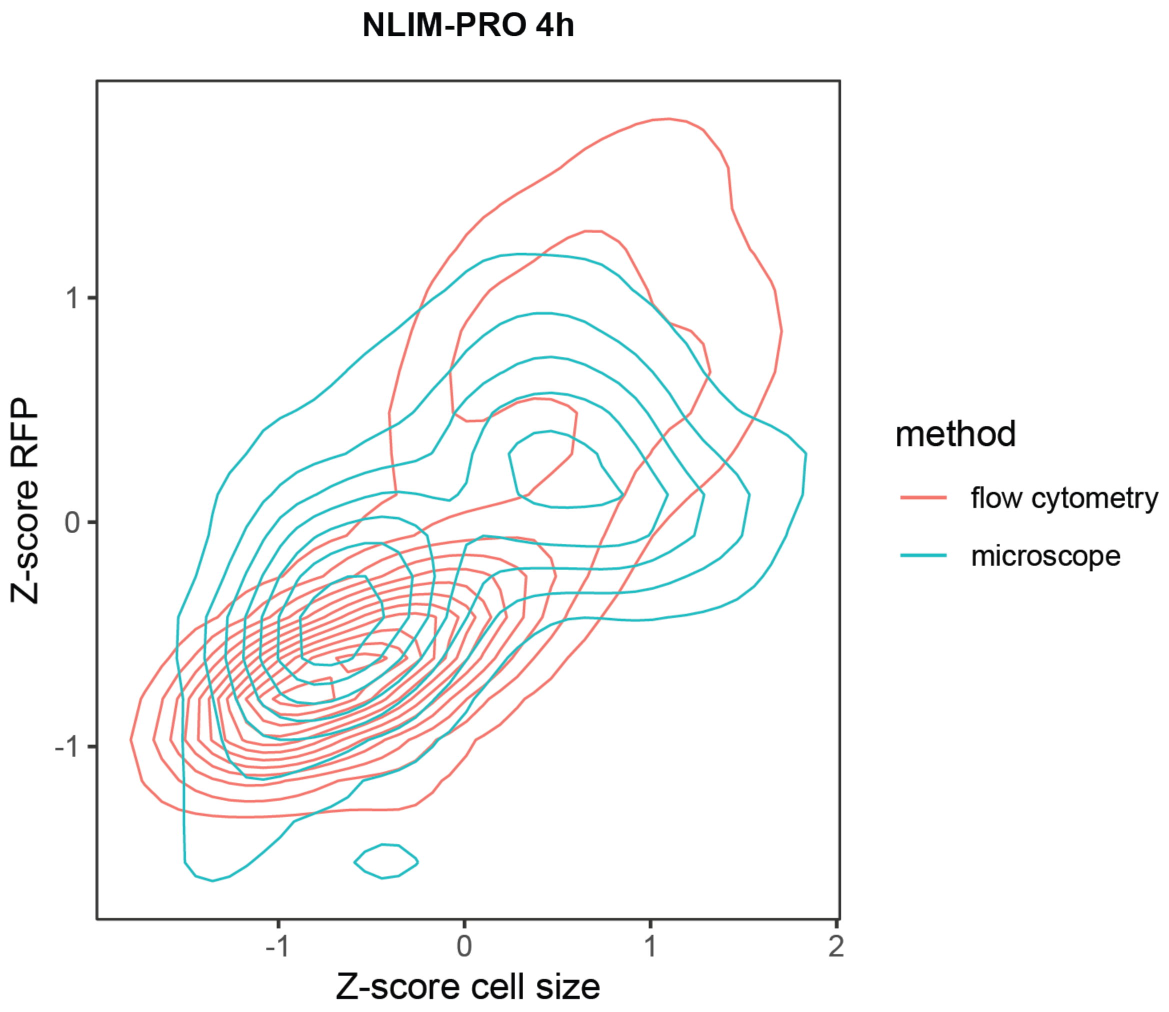

Supplementary Figure 25. Comparison between microscopy and flow cytometry for the TF library in 4h NLIM-PRO. RFP and cell size values were Z-transformed for flow cytometry and cell size and plotted due to differences in scaling.

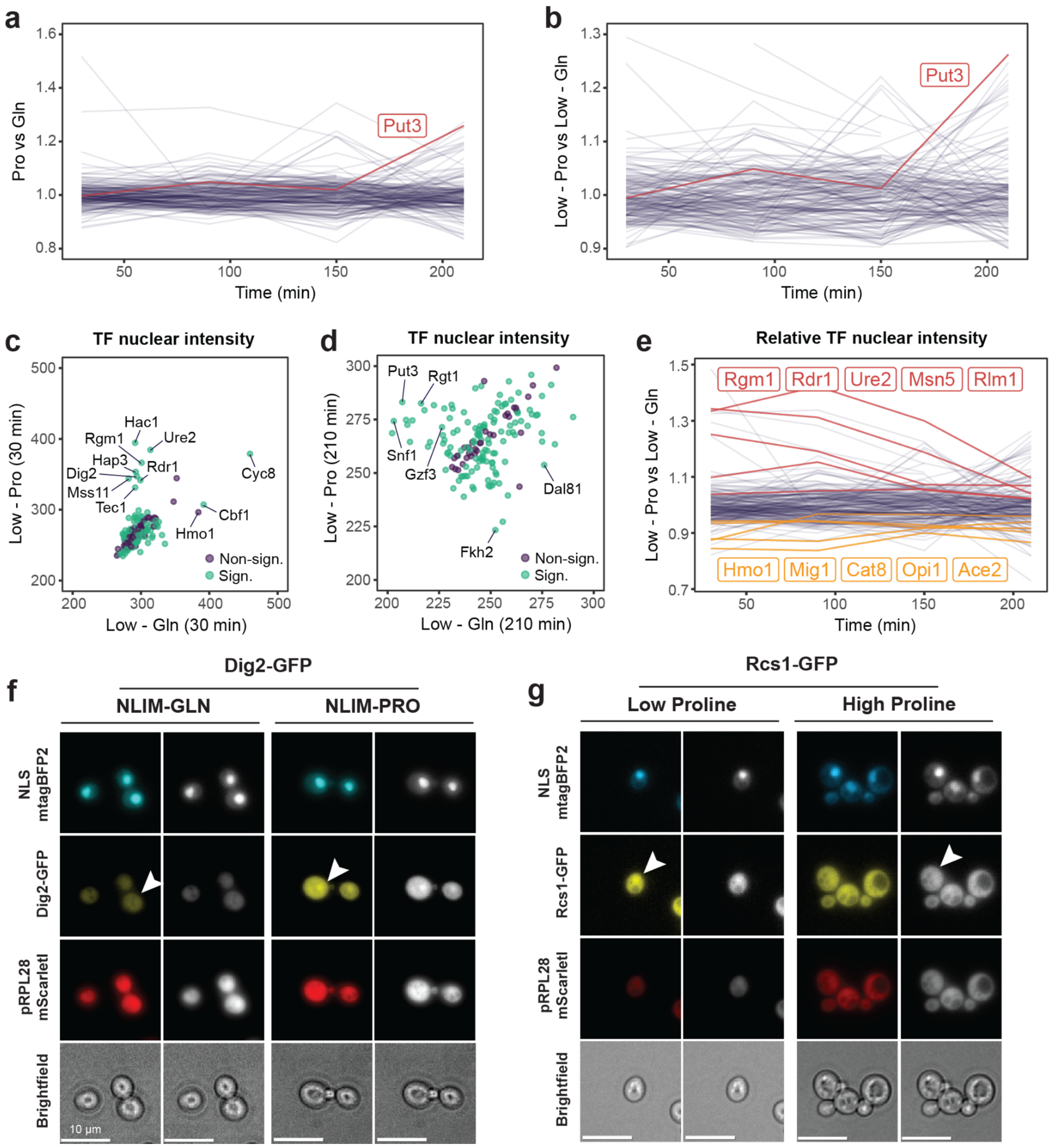

Supplementary Figure 26. Analysis of the TF library. (a, b) Relative localisation for the proline-responsive Put3 TF between bulk conditions (a) and between low proline and low glutamine subpopulation (b). **(c, d)** TF localizations for low proline plotted against low glutamine and high against low subpopulation (NLIM-PRO), 30 min (c) and 210 min (d) into the shift. TF nuclear intensity was calculated as the mean GFP fluorescence over the nucleus, determined by the NLS-mtagBFP2. **(e)** Relative TF nuclear localisation tracked over time for low proline versus low glutamine. Relative localisations were normalised by the mean relative localisation of the timepoint. **(f, g)** Multi-channel imaging of Dig2-GFP (f) and Rcs1-GFP (g). NLS mtagBFP2 represents the nuclear localisation and pRPL28-mScarletI was used to classify high and low subpopulations.

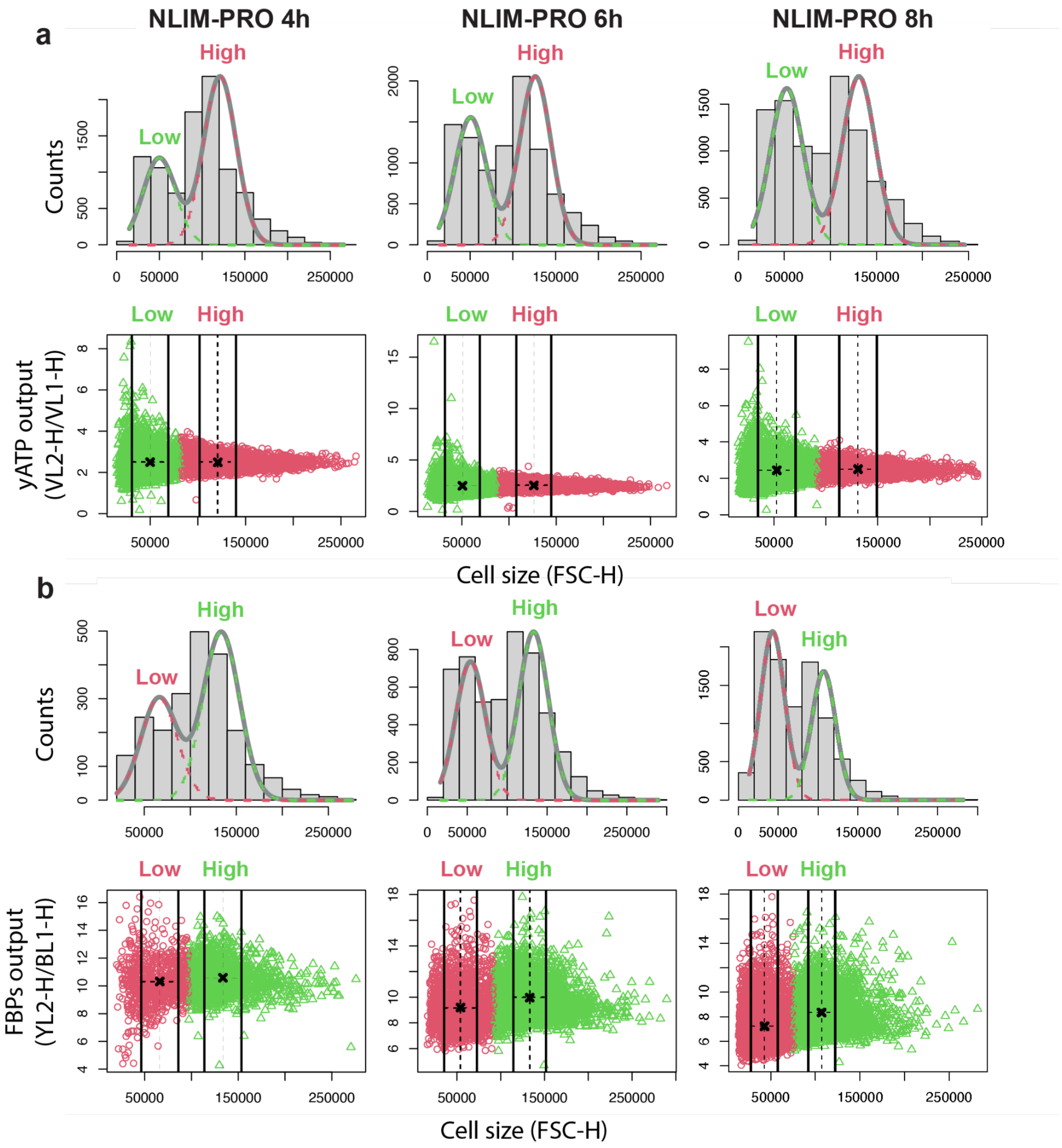

Supplementary Figure 27. Sensor output over time with Rmixmod clustering for NLIM-PRO. Results for **(a)** ATP sensor and **(b)** FBP sensor. Clustering was performed based on cell size using the R package Rmixmod.

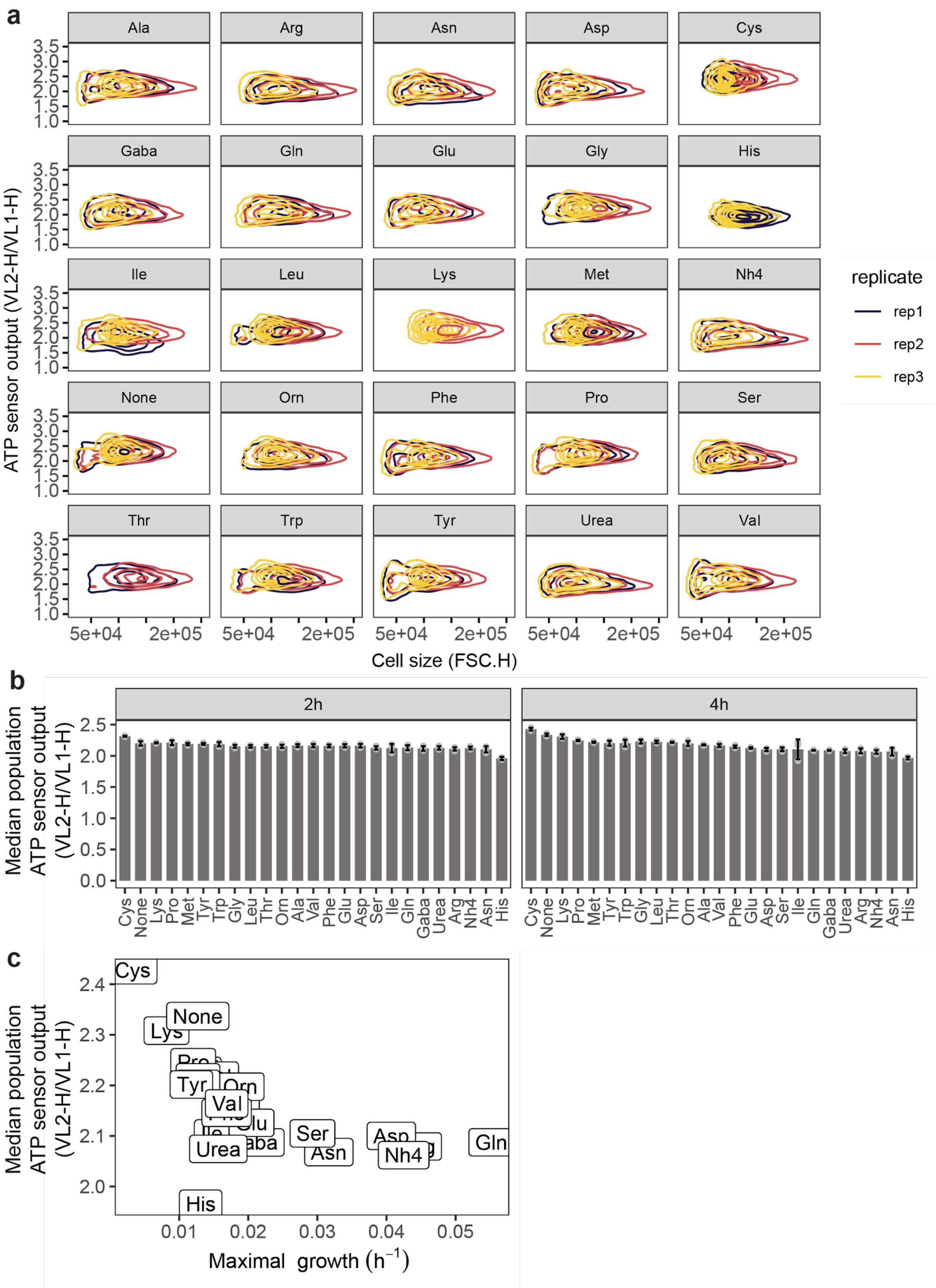

Supplementary Figure 28. ATP sensor output for all NLIM conditions. (a) Single cell ATP sensor output as a function of cell size. Samples were taken 4 h after exposure to respective conditions in triplicate experiments performed on different days. **(b)** Median population ATP sensor levels for each amino acid. **(c)** Median population ATP sensor levels (4h) versus maximal growth rate as displayed in Figure 4b. None represents the NSTARVE condition

Supplementary Note 1. High and low subpopulations are isogenic

Sorting quiescent cells can be challenging as they typically need to be distinguished from other non-growing states such as senescent cells. Consequently, sorting solely based on G1 arrest or cell density is often not sufficient.[3] In this study, we aimed to separate quiescent cells in the onset of nitrogen limitation as little as 1 hour into the shift. Flow cytometry and single-cell RNAseq analysis shows significant overlap in marker intensity and cell size, especially in the first hours into the shift (Fig. 2a, Supplementary Fig. 3). As a result, we sorted subpopulations based on both pRPL28 fluorescence (GFP FITC-A) but also cellular internal complexity as represented by the side scatter (SSC-A) (Fig. S1). Addition of the GFP marker was useful to distinguish dead cells not expressing GFP (GFP-ve). Pre-gating using FSC-H/FSC-W was used to differentiate singlets from doublets.

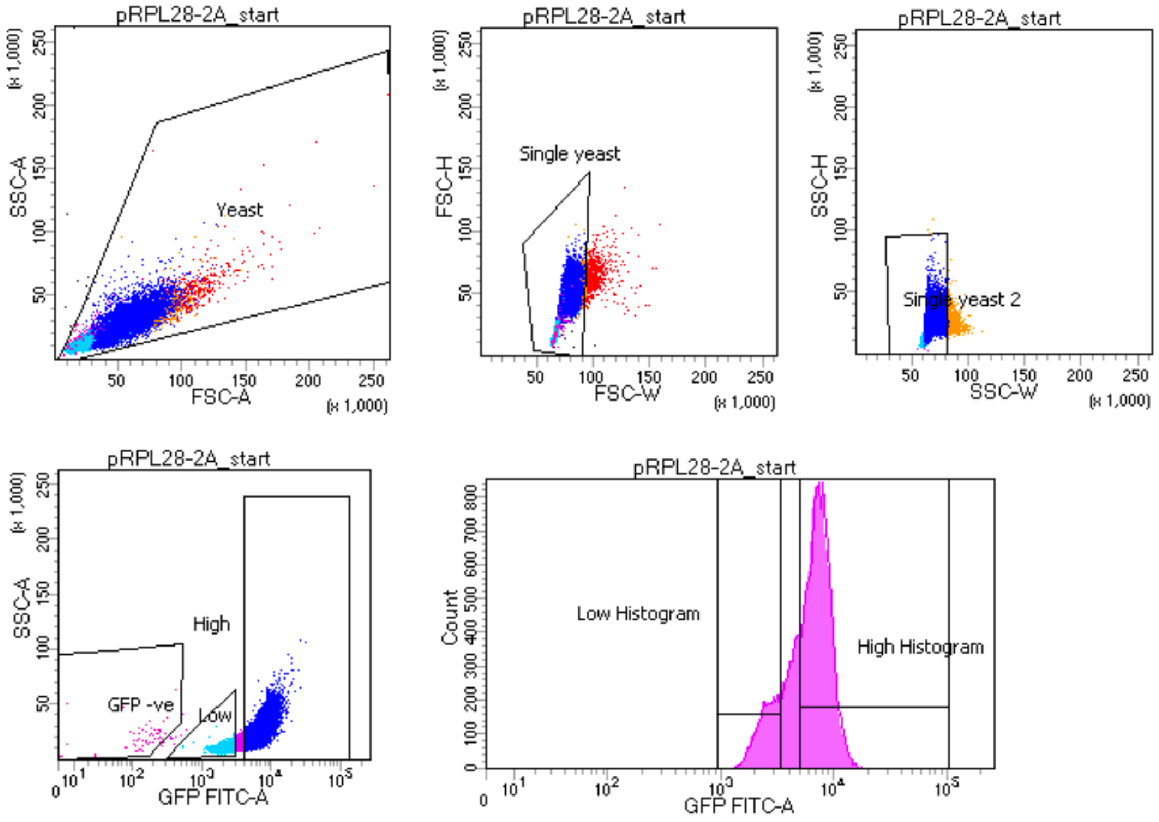

Figure S1. Typical flow cytometry data for cells exposed to a 2 h nitrogen downshift in proline media. For all sorting experiments, single yeast cells were gated according to FSC-H/FSC-W channels. Sorting of high and low populations were sorted according to SSC-A/GFP FITC-A scatter plot. We also did notice the emergence of a few cells without GFP-fluorescence (GFP -ve; <2% of the total number of cells).

To investigate if low and high subpopulations were isogenic, we sorted cells 2h after a shift from YPD to nitrogen-limited media with proline as nitrogen source (NLIM-PRO) based on pRPL28 fluorescence and side scatter followed by a recovery on YPD agar plates and flow cytometry for each sorted fraction (Fig. S2). While the GFP-negative fractions (GFP-ve) was not able to resume growth, indicating senescent cells, high and low subpopulations were able to yield high and low fractions similarly to the unsorted fraction.

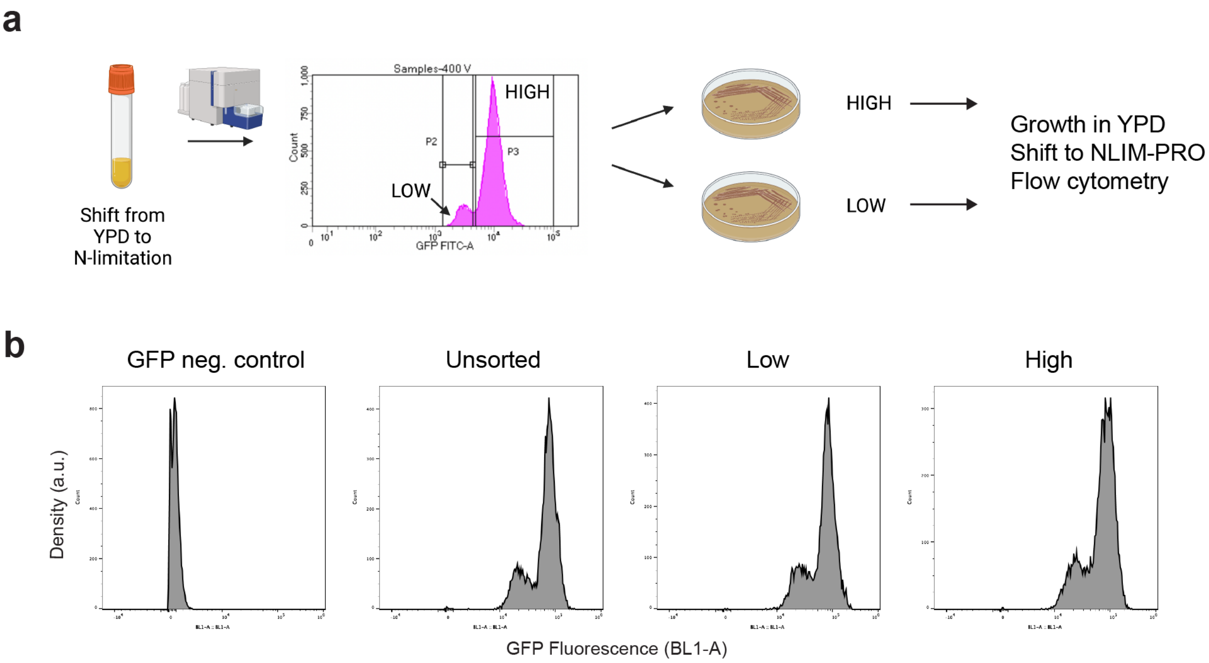

Figure S2. Low and high subpopulations are isogenic. (a) Cells exposed to a 2 h down-shift from YPD to NLIM-PRO were sorted according to GFP fluorescence (FITC-A) and cellular complexity (SSC-A) as described above. High, low and GFP-ve fraction were plated on YPD agar plate. Only high and low fractions gave colonies. For each low and high, a single colony was picked and grown overnight in YPD media. Flow cytometer and plates schematics were made with BioRender.com released under a CC-BY-NC-ND 4.0 International license. (b) Flow cytometry for sorted cultures (a) re-exposed to a 2 h shift from YPD to NLIM-PRO. Both low and high fractions show the same heterogeneity as for the unsorted fraction indicating transient isogenic heterogeneity.

Supplementary Note 2. Low subpopulation is quiescent

Despite its broad ecological importance and interesting metabolic specificity, there is currently no specific definition of quiescence other than a broad definition defining it as “a cellular state with temporary and reversible proliferation arrest[3].” In their seminal work, Klosinska et al. (2011) have shown that there are in fact many different quiescent states rather than a global one, but all quiescent states share some specificities such as resistance to stressful conditions[4]. In this supplementary note, we relate our findings with previous research on quiescence and show that the low subpopulation that emerges in both proline and glutamine has all the hallmarks of quiescence. On the temporality, we show that the low subpopulation emerges at the onset of the shift and is either maintained or relieved depending on the nitrogen source present. On the reversibility, we were also able to show through fluorescence assisted cell sorting (see Supplementary Note 1) that the low subpopulation is not in a terminal state and can resume growth on rich YPD media. Growth on YPD re-initialises the low/high status so that cells previously assigned as low can become high again.

In terms of morphology, we found that the low subpopulation is smaller than its high counterpart and cells grown in YPD which was consistent with a general cell size reduction observed in quiescent cells reported for nitrogen-starved *S. pombe* at the population level[5]. Similarly, quiescent cells are associated with a G0 state[6], where DNA content is typically in a 1N state. Flow cytometry of cells stained with propidium iodide shows that conditions leading to the highest heterogeneity in quiescence are those with an increased proportion of cells in a 1N state (Fig. S3a). For growth on proline, we also noted that this proportion was not affected by the amino acid concentration (0.8 mM vs 10 mM) but by the quality of the amino acid present. Scatter plot showed that cells with reduced DNA content were more likely those with reduced pRPL28 GFP expression and cell size (Fig. S3b).

Looking at quiescent markers, we retrieved from the NLIM-PRO scRNAseq dataset genes that were significantly different ($p_{adj} < 0.005$) between subpopulations P1 and P2. We grouped them according to which subpopulation they were more expressed and displayed their quiescence scores obtained from Klosinska et al. (Fig. S3c). We found that the low subpopulation P2 contained genes that were significantly more involved in quiescence than subpopulation P1. We also noted the up-regulation of many proteins involved in autophagy consistent with Klosinska et al. as well as up-regulation of many proteins involved in storage compounds metabolism consistent with an up-regulation of storage compounds observed in Boer *et al.*[7]

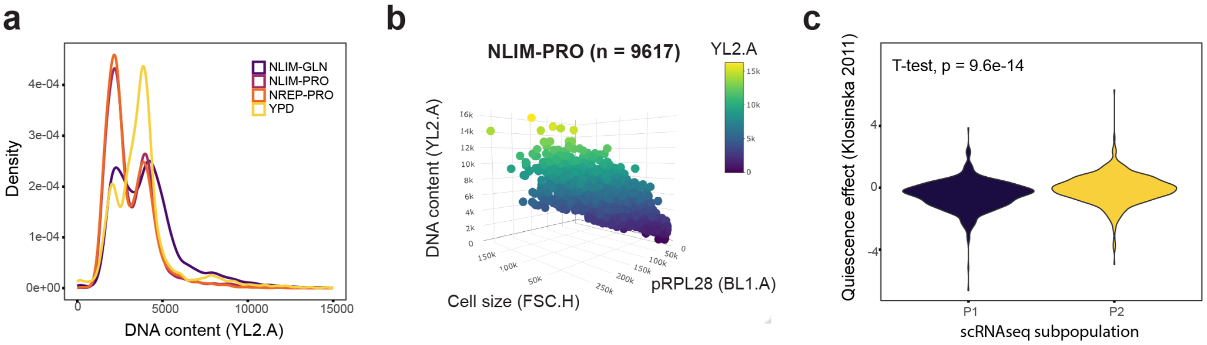

Figure S3. Low subpopulation is quiescent. (a) Flow cytometry data of pRPL28 sfGFP strains exposed to 4h down-shift and stained with propidium iodide and recorded on RFP channel (YL2-A). First peak and second peak represent 1N and 2N DNA content, respectively. (b) Flow cytometry data for NLIM-PRO condition shown in (a) with channels representing cell size (FSC-H), pRPL28 fluorescence (BL1-A) and DNA content (YL2.A). Colour indicates DNA content (YL2.A channel). (c) Quiescence scores of genes attributed to subpopulation P1 or P2 based on scRNAseq data for NLIM-PRO dataset.

Supplementary Method 1. Detailed analysis of single-cell RNAseq datasets

The dataset

We took scRNAseq data from Jackson et al.[1] The dataset contains approximately 38’000 single-cell transcriptomes of 12 transcription factor knock-outs across 11 conditions including nitrogen limitation investigated in our study. Raw data pre-processing was performed as described in the paper. The only difference from the original pre-processing was the normalisation used, where we used the default normalisation function logNormCounts from the Scuttle R package (https://github.com/LTLA/scuttle) instead of the author's custom normalisation. While this slightly changed the local shape of the subpopulations, the global structure and subpopulation clustering were similar to the original data.

Growth score calculation

Growth scores were calculated for each condition using a growth regression model mapping transcriptomic activities to growth rates[8]. Because the regression model was calibrated for bulk transcriptomics data and scales with the total number of reads for each cell in the single-cell RNAseq data, we infer relative growth scores instead of absolute predicted growth rates.

Differential expression using DESeq2 on scRNAseq datasets

DESeq2 size factors estimation does not work well on sparse count matrices that are typically associated with scRNAseq datasets. Here, we took advantage of the different genotypes (TF knockouts) present in the datasets to overcome this issue (Fig. S4a). For each gene, we summed single cell reads grouped by genotype to create each sample as input to DESeq2. For each subpopulation, this would be 12 samples representing the 12 genotypes used in Jackson et al. study. DESeq2 considers these 12 samples as technical replicates of a given subpopulation that is compared to the 12 replicates of the other subpopulation for a given condition (Fig. S4b). While pulling different genotypes together to feed DESeq2 is uncommon in bulk RNAseq analysis, we took advantage of the fact that the transcriptome variance between the two subpopulations (genotypic variance) is greater than the transcriptome variance across genotypes within a subpopulation (subpopulation variance) (Fig. S4a). The only limitation with the methodology above is to miss critical genes that significantly differ between two subpopulations but not picked up by DESeq2 because of a high variance across the different genotypes of a subpopulation.

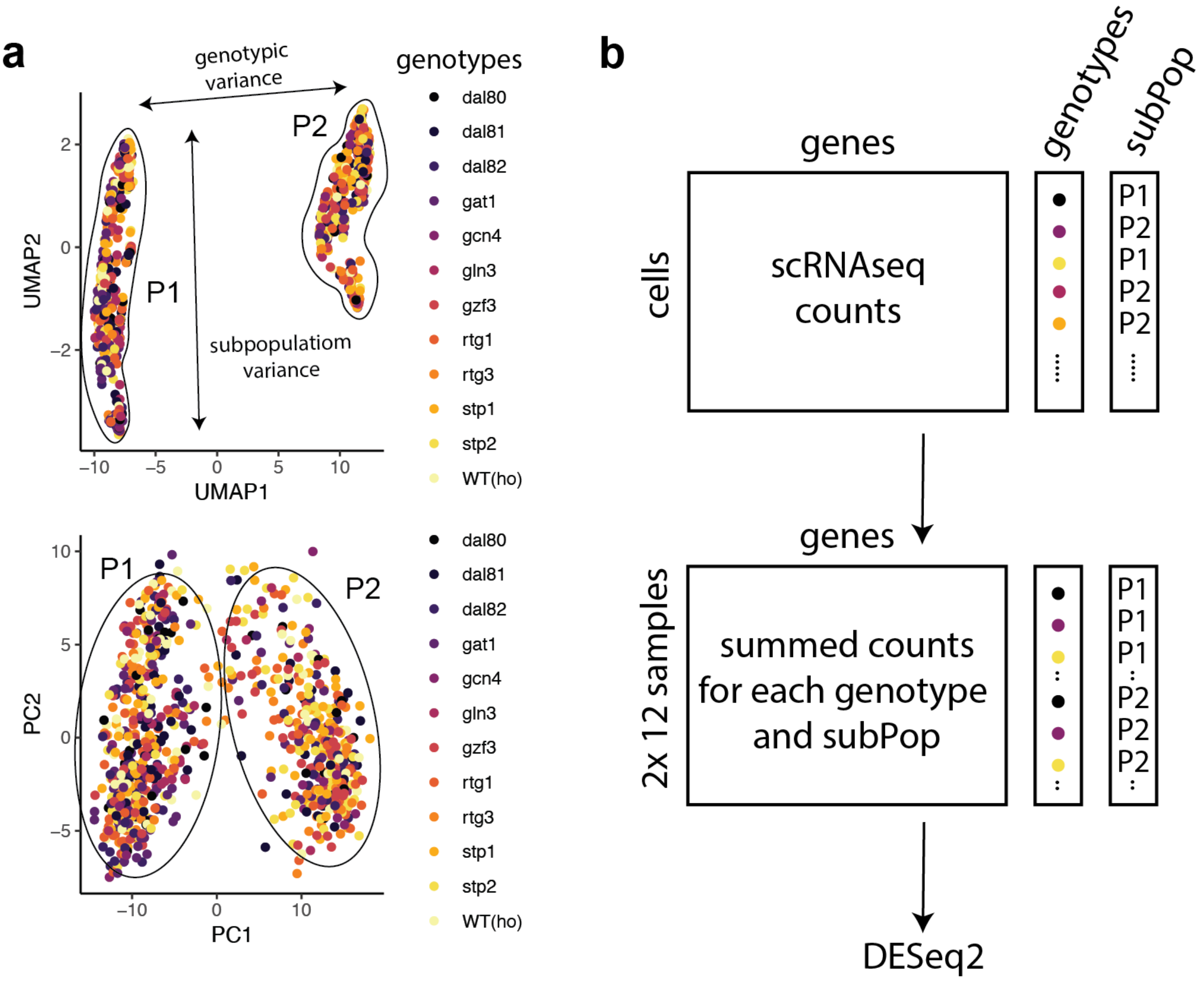

Figure S4. Single-cell RNA seq analysis workflow. (a) UMAP and PCA plots of NLIMPRO. Genotypic variance is greater than subpopulation variance. (b) DESeq2 pipeline for merging single-cell counts for a given genotype and subpopulation.

Using this methodology, DESeq2 returned 1716 and 2439 genes that were significantly different between P1 and P2 and G1 and G2, respectively (multiple hypothesis adjusted *p*-value $p < 0.005$, Benjamini-Hochberg procedure) (Supplementary Data 1). Among those, we chose the ribosomal subunit RPL28 as a subpopulation marker. It is expressed in a moderate to high amount in high and low subpopulations and directly linked to growth.

References

1. Jackson, C. A., Castro, D. M., Saldi, G.-A., Bonneau, R. & Gresham, D. Gene regulatory network reconstruction using single-cell RNA sequencing of barcoded genotypes in diverse environments. *Elife* **9**, e51254 (2020).
2. Ljungdahl, P. O. & Daignan-Fornier, B. Regulation of amino acid, nucleotide, and phosphate metabolism in Saccharomyces cerevisiae. *Genetics* **190**, 885–929 (2012).
3. Sagot, I. & Laporte, D. The cell biology of quiescent yeast - a diversity of individual scenarios. *J. Cell Sci.* **132**, (2019).
4. Klosinska, M. M., Crutchfield, C. A., Bradley, P. H., Rabinowitz, J. D. & Broach, J. R. Yeast cells can access distinct quiescent states. *Genes Dev.* **25**, 336–349 (2011).
5. Sajiki, K. *et al.* Genetic control of cellular quiescence in S. pombe. *J. Cell Sci.* **122**, 1418–1429 (2009).
6. Sun, S. & Gresham, D. Cellular quiescence in budding yeast. *Yeast* **38**, 12–29 (2021).
7. Boer, V. M., Crutchfield, C. A., Bradley, P. H., Botstein, D. & Rabinowitz, J. D. Growth-limiting intracellular metabolites in yeast growing under diverse nutrient limitations. *Mol. Biol. Cell* **21**, 198–211 (2010).
8. Airoldi, E. M. *et al.* Predicting cellular growth from gene expression signatures. *PLoS Comput. Biol.* **5**, e1000257 (2009).
