# Supplementary material for: Phenotypic heterogeneity follows a growth-viability tradeoff in response to amino acid identity
Source: Nat Commun. 2024 Aug 2;15:6515. doi: 10.1038/s41467-024-50602-8 (PMC11297284; doi:10.1038/s41467-024-50602-8)
Supplement: Supplementary file 3 — Description of Additional Supplementary Files [file 41467_2024_50602_MOESM3_ESM.pdf]

Description of Additional Supplementary Files

Supplementary Data 1. DESeq2 output for scRNAseq data analysis. (xlsx)

Supplementary Data 2. Differentially expressed genes in subpopulation RNAseq. (xlsx)

Supplementary Data 3. Strains used in this study and appearances in figures. (xlsx)

Supplementary Data 4. TF localization ranking. (xlsx)

Supplementary Data 5. Amino acid detection from supernatants of cells exposed to a 8 hours down-shift. (xlsx)
