# Supplementary material for: Phenotypic heterogeneity follows a growth-viability tradeoff in response to amino acid identity
Source: Nat Commun. 2024 Aug 2;15:6515. doi: 10.1038/s41467-024-50602-8 (PMC11297284; doi:10.1038/s41467-024-50602-8)
Supplement: Supplementary file 5 — Reporting Summary [file 41467_2024_50602_MOESM5_ESM.pdf]

nature portfolio

Corresponding author(s): Rodrigo Ledesma-Amaro
Kiyan Shabestary

Last updated by author(s): 01/07/2024

Reporting Summary

Nature Portfolio wishes to improve the reproducibility of the work that we publish. This form provides structure for consistency and transparency in reporting. For further information on Nature Portfolio policies, see our Editorial Policies and the Editorial Policy Checklist.

Statistics

For all statistical analyses, confirm that the following items are present in the figure legend, table legend, main text, or Methods section.

n/a	Confirmed	
☐	☒	The exact sample size (*n*) for each experimental group/condition, given as a discrete number and unit of measurement
☐	☒	A statement on whether measurements were taken from distinct samples or whether the same sample was measured repeatedly
☐	☒	The statistical test(s) used AND whether they are one- or two-sided<br>*Only common tests should be described solely by name; describe more complex techniques in the Methods section.*
☒	☐	A description of all covariates tested
☐	☒	A description of any assumptions or corrections, such as tests of normality and adjustment for multiple comparisons
☐	☒	A full description of the statistical parameters including central tendency (e.g. means) or other basic estimates (e.g. regression coefficient) AND variation (e.g. standard deviation) or associated estimates of uncertainty (e.g. confidence intervals)
☐	☒	For null hypothesis testing, the test statistic (e.g. *F*, *t*, *r*) with confidence intervals, effect sizes, degrees of freedom and *P* value noted<br>*Give P values as exact values whenever suitable.*
☒	☐	For Bayesian analysis, information on the choice of priors and Markov chain Monte Carlo settings
☒	☐	For hierarchical and complex designs, identification of the appropriate level for tests and full reporting of outcomes
☐	☒	Estimates of effect sizes (e.g. Cohen's *d*, Pearson's *r*), indicating how they were calculated

Our web collection on statistics for biologists contains articles on many of the points above.

Software and code

Policy information about availability of computer code

Data collection	FlowJo was used for raw flow cytometry data gating. Nikon NIS-elements was used for microscopy image aquisition. TECAN Magellan SparkControl software was used for optical density measurements.
Data analysis	All codes used for data analysis are provided freely on GitHub under https://github.com/KiyanShabestary/2023-NLIM-heterogeneity and https://github.com/Benedict-Carling/YeaZ-Output-Analysis

For manuscripts utilizing custom algorithms or software that are central to the research but not yet described in published literature, software must be made available to editors and reviewers. We strongly encourage code deposition in a community repository (e.g. GitHub). See the Nature Portfolio guidelines for submitting code & software for further information.

Data

Policy information about availability of data

All manuscripts must include a data availability statement. This statement should provide the following information, where applicable:

- Accession codes, unique identifiers, or web links for publicly available datasets
- A description of any restrictions on data availability
- For clinical datasets or third party data, please ensure that the statement adheres to our policy

Data for Fig. 1c was from Jackson et al. (2020) obtained from NCBI with accession number GSE125162. Raw sequencing data obtained for subpopulation RNAseq was deposited in NCBI GO with accession number GSE235239. All data generated or analysed during this study are included in this article and the Supplementary

Information. Source data are provided with this paper. All scripts used for data analysis and plotting are available on github (https://github.com/KiyanShabestary/2023-NLIM-heterogeneity and https://github.com/Benedict-Carling/YeaZ-Output-Analysis for HTP microscopy).

Research involving human participants, their data, or biological material

Policy information about studies with human participants or human data. See also policy information about sex, gender (identity/presentation), and sexual orientation and race, ethnicity and racism.

Reporting on sex and gender	N/A
Reporting on race, ethnicity, or other socially relevant groupings	N/A
Population characteristics	N/A
Recruitment	N/A
Ethics oversight	N/A

Note that full information on the approval of the study protocol must also be provided in the manuscript.

Field-specific reporting

Please select the one below that is the best fit for your research. If you are not sure, read the appropriate sections before making your selection.

☒ Life sciences ☐ Behavioural & social sciences ☐ Ecological, evolutionary & environmental sciences

For a reference copy of the document with all sections, see nature.com/documents/nr-reporting-summary-flat.pdf

Life sciences study design

All studies must disclose on these points even when the disclosure is negative.

Sample size	No sample size exclusion was performed. Biological replicates were used as indicated. Number of replicates were chosen based on feasibility.
Data exclusions	Due to the technical difficulty of performing subpopulation RNA sequencing after FACS sorting (time for sorting, having enough cells to perform RNA sequencing), data exclusion was based on Principal Component Analysis (shown in Extended Data Fig. 4). Samples L2_2609 and L1_2709 were clustering at two separate locations away from the main cluster (n=3) and thus not further considered when computing differentially expressed genes between high and low subpopulations.
Replication	Flow cytometry experiments were performed on different days when applicable and indicated in figure captions.
Randomization	Order of cultivations with the different nitrogen sources were random. Order of sample processing was changed between replicate experiments.
Blinding	Growth and heterogeneity measurements were semi-blinded where cultivation vials each containing a different nitrogen source were assigned a number at the start of the experiment (at least 24 different samples per run).

Reporting for specific materials, systems and methods

We require information from authors about some types of materials, experimental systems and methods used in many studies. Here, indicate whether each material, system or method listed is relevant to your study. If you are not sure if a list item applies to your research, read the appropriate section before selecting a response.

Materials & experimental systems

n/a	Involved in the study	
☒	☐	Antibodies
☒	☐	Eukaryotic cell lines
☒	☐	Palaeontology and archaeology
☒	☐	Animals and other organisms
☒	☐	Clinical data
☒	☐	Dual use research of concern
☒	☐	Plants

Methods

n/a	Involved in the study	
☒	☐	ChIP-seq
☐	☒	Flow cytometry
☒	☐	MRI-based neuroimaging

Flow Cytometry

Plots

Confirm that:

☒ The axis labels state the marker and fluorochrome used (e.g. CD4-FITC).

☒ The axis scales are clearly visible. Include numbers along axes only for bottom left plot of group (a 'group' is an analysis of identical markers).

☒ All plots are contour plots with outliers or pseudocolor plots.

☒ A numerical value for number of cells or percentage (with statistics) is provided.

Methodology

Sample preparation	Flow cytometry was used for analysis and sorting. In both cases, flow cytometry was performed on different days when indicated. Biological replicates were resuspended in PBS (from 1:2 to 1:10 depending on OD) prior to analysis/sorting.
Instrument	For analysis, an Attune Nxt flow cytometer (Invitrogen) was used. For sorting, a BD FACSAria III Cell Sorter was used.
Software	For the Attune Nxt flow cytometer, Attune Cytometric software was used for data collection. For the BD FACSAria III Cell Sorter, BD FACSDiva software was used for data collection and subpopulation gating.
Cell population abundance	The low subpopulation was present in our samples at abundances depending on post-shift timing, ranging from 5% to 80% of the total population. For the sorting runs, purity was performed by re-running each post-sort fraction. Purity was 98% for the low fraction and 90% for the high fraction (due to cells continuing to differentiate from high to low during the time elapsed (5 mins) between sort and purity runs).
Gating strategy	For sorting, subpopulations were sorted based on both pRPL28 fluorescence (GFP FITC-A) but also cellular internal complexity as represented by the side scatter (SSC-A). GFP FITC-A was used to distinguish dead cells not expressing GFP (GFP-ve). Pre-gating using FSC-H/FSC-W was used to differentiate singlets from doublets. Gating strategy and plots are shown in Supplementary Note 1. For analysis, doublets exclusion were performed using FSC-H/FSC-A. For cell viability measurements using Attune YL1-H channel, viability was thresholded based on viability measured in YPD during exponential growth.

☒ Tick this box to confirm that a figure exemplifying the gating strategy is provided in the Supplementary Information.
